# Impact of soluble epoxide hydrolase inhibition on silica-induced pulmonary fibrosis, ectopic lymphoid neogenesis, and autoantibody production in lupus-prone mice

**DOI:** 10.1080/08958378.2024.2413373

**Published:** 2024-10-17

**Authors:** Olivia F. McDonald, James G. Wagner, Ryan P. Lewandowski, Lauren K. Heine, Vanessa Estrada, Elham Pourmand, Megha Singhal, Jack R. Harkema, Kin Sing Stephen Lee, James J. Pestka

**Affiliations:** a Department of Pharmacology and Toxicology, College of Osteopathic Medicine, Michigan State University, East Lansing, MI, USA; b Institute for Integrative Toxicology, Michigan State University, East Lansing, MI, USA; c Department of Microbiology, Genetics, and Immunology, Michigan State University, East Lansing, MI, USA; d Department of Pathobiology and Diagnostic Investigation, Michigan State University, East Lansing, MI, USA; e Los Alamos National Laboratory, Los Alamos, NM, USA; f Department of Chemistry, Michigan State University, East Lansing, MI, USA; g Department of Food Science and Human Nutrition, Michigan State University, East Lansing, MI, USA

**Keywords:** Pulmonary fibrosis, crystalline silica, lung, NZBWF1 mice, soluble epoxide hydrolase, ectopic lymphoid structure, prostaglandin

## Abstract

**Objective:**

Acute intranasal (IN) instillation of lupus-prone NZBWF1 mice with crystalline silica (cSiO_2_) triggers robust lung inflammation that drives autoimmunity. Prior studies in other preclinical models show that soluble epoxide hydrolase (sEH) inhibition upregulates pro-resolving lipid metabolites that are protective against pulmonary inflammation. Herein, we assessed in NZBWF1 mice how acute IN cSiO_2_ exposure with or without the selective sEH inhibitor TPPU influences lipidomic, transcriptomic, proteomic, and histopathological biomarkers of inflammation, fibrosis, and autoimmunity.

**Methods:**

Female 6-week-old NZBWF1 mice were fed control or TPPU-supplemented diets for 2 weeks then IN instilled with 2.5 mg cSiO_2_ or saline vehicle. Cohorts were terminated at 7 or 28 days post-cSiO_2_ instillation (PI) and lungs analyzed for prostaglandins, cytokines/chemokines, gene expression, differential cell counts, histopathology, and autoantibodies.

**Results:**

cSiO_2_-treatment induced prostaglandins, cytokines/chemokine, proinflammatory gene expression, CD206^+^ monocytes, Ly6B.2^+^ neutrophils, CD3^+^ T cells, CD45R^+^ B cells, centriacinar inflammation, collagen deposition, ectopic lymphoid structure neogenesis, and autoantibodies. While TPPU effectively inhibited sEH as reflected by skewed lipidomic profile in lung and decreased cSiO_2_-induced monocytes, neutrophils, and lymphocytes in lung lavage fluid, it did not significantly impact other biomarkers.

**Discussion:**

cSiO_2_ evoked robust pulmonary inflammation and fibrosis in NZBWF1 mice that was evident at 7 days PI and progressed to ELS development and autoimmunity by 28 days PI. sEH inhibition by TPPU modestly suppressed cSiO_2_-induced cellularity changes and pulmonary fibrosis. However, TPPU did not affect ELS formation or autoantibody responses, suggesting sEH minimally impacts cSiO_2_-triggered lung inflammation, fibrosis, and early autoimmunity in our model.

## Introduction

Occupational exposure to respirable crystalline silica (cSiO_2_) has been etiologically linked to pulmonary fibrosis, lung cancer, and systemic autoimmune diseases like lupus (Pollard 2016; Hoy and Chambers 2020; Boudigaard et al. 2021). Excessive cSiO_2_ exposure occurs in dusty occupations including mining, construction, ceramics, and dentistry work (Salamon et al. 2021; Wiebert et al. 2022; Misra et al. 2023). In female lupus-prone mice, we have previously demonstrated that 4 weekly intranasal (IN) instillations with 1 mg cSiO_2_, modeling one half of human lifetime exposure at the exposure limit recommended by NIOSH, triggers in the lung upregulation of proinflammatory mediators (i.e. cytokines, chemokines, interferons, adhesion molecules) and genes involved with innate and adaptive immune cell function. This occurs in as little as 1 week post-instillation (PI) with further upregulation up to 13 weeks PI (Bates et al. 2019). Repeated cSiO_2_ instillations also elicit the development of ectopic lymphoid structures (ELS) in the lung and consequent elevations in diverse autoantibodies (AAb) in the BALF and plasma that hasten glomerulonephritis and mortality (Bates et al. 2015, 2018; Rajasinghe et al. 2020).

To model immediate and short-term effects of cSiO_2_ on inflammation and early autoimmunity in the lung, we recently assessed the effects of a singular IN dose of 2.5 mg cSiO_2_ in female NZBWF1 mice on cellular, histopathological, transcriptomic, and protein biomarkers from 1 to 28 days PI. We found in this model of acute cSiO_2_ toxicity that the particle evokes robust inflammation in the lung by 7 days PI, characterized by (i) alveolar infiltration of macrophages, neutrophils, and lymphocytes, (ii) cell death and release of cellular dsDNA, (iii) upregulation of proinflammatory cytokines, chemokines, and type I interferon (IFN)-regulated genes, and (iv) secretion of proinflammatory cytokines and chemokines. Further apparent was the development of T and B lymphocyte-containing ELS in the lung beginning at 21 days PI, indicative of preliminary development of cSiO_2_-induced autoimmunity (Chauhan et al. 2021). Taken together, this novel model may provide valuable insight into early mechanisms by which cSiO_2_ drives inflammation and fibrosis in the lung, as well as provide a preclinical model for evaluating potential interventions against environmentally triggered lung disease.

While environmental toxicants, such as cSiO_2_, can potentiate the development of lung disease, other environmental factors, such as dietary polyunsaturated fatty acids (PUFAs), can also influence disease onset. In the United States, daily intake of ω–6 PUFAs exceeds that of ω–3 PUFAs at a ω–6/ω–3 ratio of 20:1 and is associated with an increased risk of inflammatory disease (Forsyth et al. 2016; Innes and Calder 2018; DiNicolantonio and O’Keefe 2021). Consumed ω–3/6 PUFAs incorporate into the cell membrane and consequently serve as substrates for potent proinflammatory and pro-resolving lipid mediators. One of the most important cell membrane PUFAs in inflammatory signaling is arachidonic acid (C20:4, ω–6, ARA) (Innes and Calder 2018). Phospholipase A2 (PLA2), when activated by an inflammatory stimulus, cleaves ARA from the sn-2 position of cellular phospholipids. Released non-esterified ARA can be shunted into one of three major eicosanoid biosynthesis pathways: (i) the cyclooxygenase (COX) pathway which converts ARA into prostaglandins and thromboxanes; (ii) the lipoxygenase (LOX) pathway which converts ARA into leukotrienes; and (iii) the cytochrome P450 (CYP450) pathway which converts ARA into epoxyeicosatrienoic acids (EpETrEs). Generally, prostaglandins, thromboxanes, and leukotrienes are considered proinflammatory, whereas EpETrEs are considered pro-resolving (Calder 2020). In patients with idiopathic pulmonary fibrosis (IPF) and preclinical models of pulmonary fibrosis, it has been noted that (i) LTB4 and PGE2 are increased in lung tissue, (ii) 11,12-EpETrE is decreased in lung tissue, (iii) PGE2 is decreased in BALF, and (iv) PGF2α is increased in plasma—all of which are suggestive of dysregulated ARA metabolism (Aihara et al. 2013; Bärnthaler et al. 2020; Kim et al. 2021; Pang et al. 2021; Wygrecka et al. 2023).

One possible intervention for delaying the development and progression of environmentally triggered lung disease is pharmacological modification of the endogenous lipidome. Soluble epoxide hydrolase (sEH) is a promising drug target that converts highly pro-resolving CYP450-derived EpETrEs to less potent dihydroxyeicosatrienoic acids (DiHETrEs) (Wagner et al. 2017; Iyer et al. 2022). In preclinical rodent studies, the sEH inhibitor (sEHI) 1-(4-trifluoro-methoxyphenyl)-3-(1-propionylpiperidin-4-yl) urea (TPPU) has been reported to ameliorate ongoing inflammation and fibrosis in multiple organs including the lung and kidney (Guan et al. 2021; Shi et al. 2022) and various autoimmune conditions (Trindade-da-Silva et al. 2017; Klocke et al. 2019; Jonnalagadda et al. 2021). TPPU has an excellent pharmacological profile characterized by high oral bioavailability, affinity for sEH, and biological potency and minimal non-specific binding and adverse side-effects (Rose et al. 2010; Liu et al. 2013; Lee et al. 2014; Ostermann et al. 2015). Recently, we have demonstrated that oral administration of TPPU in an experimental rodent diet increases the plasma epoxide/diol metabolite ratio, and, furthermore, TPPU ameliorates lipopolysaccharide (LPS)-accelerated kidney damage in female NZBWF1 mice (Favor et al. 2023). Thus, TPPU may be efficacious in ameliorating inflammation, fibrosis, and comorbidities triggered by other environmental agents like cSiO_2_.

The objective of our present study was to test the hypothesis that sEH inhibition will suppress acute cSiO_2_-induced pulmonary fibrosis and autoimmunity in lupus-prone NZBWF1 mice. Female NZBWF1 mice were used because they express genetic loci that contribute to increased autoreactive T and B cell numbers, elevated B cell hyperactivity, and reduced T cell death (Borchers et al. 2000). These aberrations culminate in elevated antinuclear AAb titers, loss of immunological self-tolerance, and spontaneous development of systemic autoimmune disease similar to lupus in humans (Raveche et al. 1980; Borchers et al. 2000).

## Materials and methods

### Key reagents

All key reagents and their corresponding catalog numbers are listed in Supplementary Table 1.

### Diets

Two experimental diets were prepared using a modified American Institute of Nutrition (AIN)-93G diet (Dyets Inc., Bethlehem, PA, USA) containing 70 g/kg fat to provide optimal nutrition to experimental rodents (Reeves et al. 1993). Both CON and TPPU diets contained 60 g/kg high-oleic safflower oil (Hain Pure Food, Boulder, CO, USA) and 10 g/kg corn oil for essential ω–9 and ω–6 fatty acids, respectively. TPPU diet included 22.5 mg TPPU, which was synthesized by our lab using a published protocol (Rose et al. 2010) and mixed into 1 kg of CON diet. To prevent lipid oxidation, experimental diets were prepared biweekly and stored at −20 °C until administered to mice. A fresh diet was given to mice every day. Diet formulations are recorded in Table 1.

### Animals

**Figure 1.**
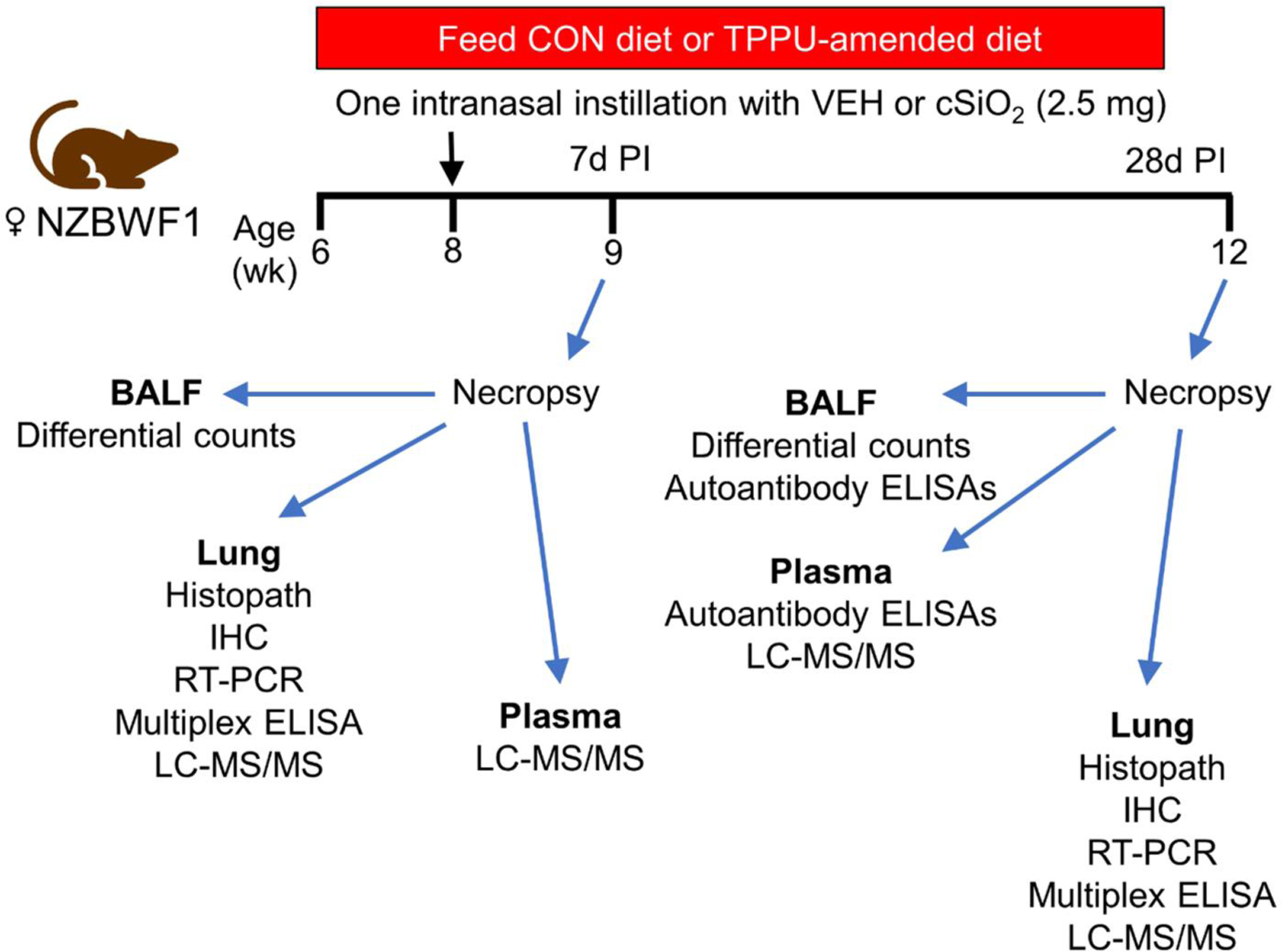
Experimental design. At 6 weeks of age, female lupus-prone NZBWF1 mice (*n=*48) were fed either control (CON) diet or TPPU-supplemented diet. Upon reaching 8 weeks of age, mice were intranasally instilled with 25 ml of saline vehicle (VEH) or 2.5 μg of cSiO_2_ suspended in 25 μl of saline. Cohorts of mice were sacrificed at 9 weeks of age (7d post-instillation [PI]) and 12 weeks of age (28d PI). Lung tissue, bronchoalveolar lavage fluid (BALF), and plasma were collected for downstream analyses.

All experimental protocols were approved by the Michigan State University (MSU) Institutional Animal Care and Use Committee (Animal Use Form [AUF] #PROTO202100252) in accordance with National Institutes of Health guidelines. Female lupus-prone NZBWF1 mice (cat. #100008) aged 6 weeks were procured from the Jackson Laboratory (Bar Harbor, ME, USA) and randomized into experimental groups (Table 2). Mice were housed 4 per cage and given free access to drinking water and experimental diet. Animal facilities were maintained under controlled conditions (lighting: 12 h light/dark cycle; temperature: 21–24 °C; humidity: 40–55%). Mice were given 2 weeks to acclimate before experiments began (Figure 1).

### Intranasal cSiO_2_ instillation

At 8 weeks of age, mice were IN instilled once with 2.5 mg cSiO_2_ as described previously (Chauhan et al. 2021). Briefly, acid-washed, oven-dried cSiO_2_ particles (Min-U-Sil^®^ 5, average particle size: 1.5–2.0 μm, Pennsylvania Sand Glass Corporation, Pittsburgh, PA, USA) were suspended in sterile phosphate buffered saline (PBS; Millipore Sigma) at 100 mg/ml before use. cSiO_2_ stock suspensions were sonicated and vortexed vigorously before use. Mice were anesthetized by inhalable isoflurane (4% in O_2_), held in the supine position, and instilled with either 2.5 mg cSiO_2_ suspended in 25 μl PBS or 25 μl PBS vehicle (VEH). This dose was chosen because it has been widely used in silicosis studies (Re et al. 2014; Nakashima et al. 2018; Zhao et al. 2020; Kumari and Singh 2022) and allometrically reflects 30% of lifetime human occupational exposure to respirable cSiO_2_ at the permissible exposure limit (PEL) of 50 μg/m^3^/day defined by the U.S. Occupational Safety and Health Administration (OSHA 2019). Mice were held in the same position for a few seconds after instillation to ensure adequate distribution throughout the respiratory tract, then mice were returned to their cages and monitored for signs of distress. No injury or death resulted from the procedure. Cohorts of VEH- and cSiO_2_-instilled mice (3 groups, *n=*8/group) were terminated at days 7 and 28 PI. These endpoints were selected because acute cSiO_2_ instillation was previously found to elicit robust pulmonary leukocyte recruitment, chemokine and interferon-regulated gene expression, cell death, and AAb secretion at both 7 and 28 days PI (Chauhan et al. 2021).

### Necropsy tissue collection and processing

Mice were euthanized by intraperitoneal injection of 56 mg/kg sodium pentobarbital and subsequent abdominal aortic exsanguination. Blood was immediately collected with heparin-coated syringes and centrifuged at 3500 ×*g* for 10 min at 4 °C to isolate plasma. An antioxidant cocktail (0.2 mg/mL butylated hydroxytoluene, 0.2 mg/mL triphenylphosphine, 0.6 mg/mL EDTA) (Rand et al. 2018) was prepared in-house and added at a 5% (v/v) concentration to plasma aliquots designated for LC-MS/MS analysis. Plasma samples were stored at −80 °C as single-use aliquots for downstream analyses. After blood collection, the trachea was exposed and cannulated, and the lungs and heart were collected *en bloc*. Isolated lungs were flushed twice with 0.8 ml of sterile PBS through the cannulated trachea to recover bronchoalveolar lavage fluid (BALF), and BALF fractions were combined for downstream analyses. Cranial, middle, and accessory lobes were removed, snap-frozen in liquid nitrogen, and stored at −20 °C. The caudal lobe was stored in RNAlater (Thermo Fisher Scientific, Waltham, MA, USA) overnight at 4 °C then stored at −80 °C for RNA analysis. The left lung lobe was intratracheally fixed with 10% (v/v) neutral-buffered formalin (Fisher Scientific, Pittsburgh, PA, USA) at a constant pressure (30 cm H_2_O) for 1 h and subsequently immersed and stored in a large volume of 10% formalin for 24 h. All fixed tissues were transferred to 30% (v/v) ethanol for long-term storage and histological preparation.

### Homogenization of lung tissue for LC-MS/MS analysis

Lung tissues were weighed and placed in low-binding vials containing a proportional volume of PBS, 2% (v/v) in-house antioxidant cocktail (Rand et al. 2018), and 5–6 homogenization beads. To each vial, 10 μM of the sEH inhibitor TPCU was added, then vials were briefly vortexed and snap-frozen in liquid nitrogen. A Precellys Evolution Touch tissue homogenizer (Bertin Technologies, Rockville, MD, USA) was set to 6800 rpm, and tissues were homogenized by running six 20-second cycles with 30-second pauses between each cycle. Afterward, vials were centrifuged at 10000 rpm for 10 min at 4 °C, and resulting lung homogenates were kept on ice for solid-phase extraction (SPE).

### Solid-phase extraction (SPE) of TPPU and selected oxylipins from lung homogenate and plasma

Waters Oasis-HLB SPE cartridges (part #WAT094226) were used for SPE as previously described (Favor et al. 2023). Briefly, cartridges were first washed once with 2 ml of ethyl acetate, twice with 2 ml of methanol, and twice with 2 ml of 95:5 (v/v) water/methanol + 0.1% (v/v) acetic acid, then plasma or lung homogenate samples were applied to separate cartridges. Before samples moved into the cartridge sorbent, 10 μl of deuterated internal standard solution (16 nM BGB2-d4, 10 nM LTB4-d4, 16 nM 8,9-DiHETrE-d11, 16 nM 9-HODE-d4, 20 nM 15(S)-HETE-d8, 40 nM 5(S)-HETE-d8, 40 nM 8,9-EpETrE-d11) was directly added into the samples. Cartridges were washed with 1.5 ml of 95:5 (v/v) water/methanol + 0.1% (v/v) acetic acid, then water and unwanted residues were removed using a low vacuum for 20 min. Afterward, analytes were eluted by adding 0.5 ml of methanol followed by 1 ml of ethyl acetate to the cartridges then transferred into separate low-binding vials containing 6 μl of elution trap solution (30% [v/v] glycerol in methanol). A high vacuum was then used for 1–2 h to evaporate residual solvent in analyte eluents, and concentrated residues were reconstituted in 100 μl of 10 nM 12-[[(cyclohexylamino)carbonyl]amino]-dodecanoic acid (CUDA) internal standard in 75% (v/v) ethanol. Samples were then vortexed for 10 min, passed through a 0.45-lm filter, transferred to high-performance liquid chromatography (HPLC) vials with salinized inserts, purged with argon gas, and stored at −80 °C until LC-MS/MS analysis.

### LC-MS/MS quantitation of TPPU and selected oxylipins in lung homogenate and plasma

Ultra-performance liquid chromatography (UPLC) was performed using a Waters ACQUITY UPLC CSH^™^ C18 2.1 × 150 mm, 1.7 μm UPLC column (part #186005298, ser. #01653023815121) connected to a Waters Xevo TQ-XS tandem quadrupole UPLC/MS/MS spectrometer (Milford, MA, USA) at the MSU Mass Spectrometry and Metabolomics Core. The mass spectrometer was equipped with a Waters ACQUITY SDS pump, Waters ACQUITY CM detector, and Waters ACQUITY Sample Manager FTN-1 held at 10 °C during sample injection (Milford, MA, USA). A previously described UPLC chromatographic method (Favor et al. 2023) was used to separate analytes within each sample. Briefly, mobile phase A consisted of 0.1% (v/v) acetic acid in water and mobile phase B consisted of 84:16 (v/v) acetonitrile/methanol + 0.1% (v/v) glacial acetic acid during gradient elution. The flow rate was set to 250 μl/min, run time was set to 20 min, and sample volume was set to 10 μl (Supplementary Table 2).

Negative electrospray served as the ionization source for multiple reaction monitoring (MRM) modes. An 8spots-calibration linear standard curve was established using deuterated fatty acid metabolite standards to quantify sample oxylipins, and a separate 8spots-calibration standard curve was established using TPPU to quantify sample TPPU concentration. Standard curves were used by Waters MassLynx^™^ MS software v4 (Milford, MA, USA) to calculate analyte area, internal standard (IS) area, raw concentration (in nM), and signal-to-noise (S/N) ratio for each analyte in all plasma and lung samples. For plasma samples, analyte concentrations were normalized by dividing raw concentrations by 2, as SPE concentrated the analytes by a factor of 2. For lung homogenate samples, normalized analyte concentrations were calculated by dividing raw analyte concentrations by the mass of lung tissue per sample homogenized.

### Expression of inflammatory cytokine, chemokine, and type I interferon-regulated genes in the lung

Total RNA from the lung was extracted using TissueLyser II (Qiagen, Germantown, MD, USA) and an RNeasy Mini Kit (Qiagen) according to the manufacturer’s instructions. Isolated RNA was reconstituted in RNase-free water and quantified using a Nanodrop ND-1000 spectrophotometer (Thermo Fisher Scientific). RNA was reverse transcribed at a concentration of 100 ng/μl using a High-Capacity cDNA Reverse Transcriptase Kit (Thermo Fisher Scientific). Taqman assays for proinflammatory cytokines (*Il1a*, *Il1b*, *Tnf*), chemokines (*Ccl2*, *Cxcl5*, *Cxcl10*), type I interferon-related genes (*Irf7*, *Mx1*, *Oas2*), and endogenous housekeeping genes (*Actb*, *Gapd*, *Hprt*) were run with technical triplicates using a Smart Chip Real-Time PCR System at the MSU Genomics Core. Expression levels of selected genes of interest were normalized to housekeeping genes and reported as fold-change compared to the VEH/CON group using the 2^−ΔΔCT^ method (Vandesompele et al. 2002).

### Profiling of proinflammatory cytokines and chemokines in the lung

Lung tissues were weighed and homogenized in RIPA Lysis and Extraction Buffer (Thermo Fisher Scientific) using TissueLyser II (Qiagen) to yield 20% (w/v) homogenate in buffer. Total protein in each sample was quantified using a Pierce^™^ BCA Protein Assay Kit (Thermo Fisher Scientific) and sample absorbances measured using a FilterMax F3 Multimode plate reader (Molecular Devices, San Jose, CA, USA) set to 562 nm. Samples were normalized to a total protein concentration of 1000 μg/ml by adding the appropriate volume of RIPA buffer. Then, 100-μl sample aliquots were shipped to Eve Technologies (Calgary, AB, Canada) for quantification of homogenate cytokines and chemokines using Mouse Cytokine/Chemokine 44-Plex Discovery Assay^®^ Array. Resultant cytokine and chemokine levels were normalized to the original weight of lung tissue homogenized per animal and reported in units of pg/g lung tissue.

### BALF inflammatory cell quantitation

Total cells in BALF were determined by counting intact cells with a standard hemocytometer. Cytological slides were prepared by centrifuging 150 μl of BALF from each mouse onto microscopic slides at 600 × *g* for 10 min using a Shandon Cytospin 3 (Shandon Scientific, PA, USA), drying overnight at 25 °C, and staining with Diff-Quick (Thermo Fisher Scientific). Differential counts of monocytes/macrophages, neutrophils, eosinophils, and lymphocytes were determined by assessing the morphological criteria of 200 counted cells on each slide.

### Lung histopathology, immunofluorescence, and birefringent imaging

Formalin-fixed left lung lobes were cut into 5-mm sections, embedded in paraffin, then deparaffinized and stained with hematoxylin and eosin (H&E) or Masson’s trichrome at the MSU Investigative Histopathology Laboratory. Tissues stained with H&E were microscopically imaged and semi-quantitatively graded for the following lesions: (a) presence of centriacinar inflammation, (b) presence of centriacinar fibrosis, and (c) presence of perivascular lymphoid cells. Each lung was microscopically evaluated by a board-certified veterinary pathologist who did not have previous knowledge of individual animal exposure or treatment history (i.e. ‘blinded analysis’). Semi-quantitative analysis of pulmonary histopathology and collagen deposition used the following criteria: (0) no changes compared to control mice, (1) minimal (<10% of total area affected); (2) slight (10–25% of total area affected), (3) moderate (26–50% change affected), (4) severe (51–75% of total area affected), or (5) very severe (>75% of total area affected).

Immunohistochemical identification of neutrophils, monocytes, B lymphocytes, and T lymphocytes in the lung was performed as previously described (Bates et al. 2015). Briefly, H&E-stained lung sections were stained with mouse-specific anti-Ly6B.2 monoclonal antibody (BioRad, Hercules, CA, USA) for neutrophil detection, anti-CD206 polyclonal antibody (Abcam, Cambridge, MA, USA) for monocyte detection, anti-CD45R monoclonal antibody (Becton Dickinson, Franklin Lakes, NJ, USA) for B lymphocyte detection, or anti-CD3 polyclonal antibody (Abcam) for T lymphocyte detection. Slides were scanned with an Olympus VS200 virtual slide scanner (Evident Scientific & Olympus VS200, Waltham, MA, USA) equipped with a UPLXAPO 20X objective lens (Olympus) and a VS-264C RGB camera (IDS Imaging Development Systems Inc., Stoneham, MA, USA). Semi-quantitative scores for neutrophil/macrophage infiltration and perivascular/peribronchiolar lymphocyte aggregation in the lung were assigned using the following scale: (0) no changes compared to VEH/CON mice, (1) minimal (<10% affected), (2) mild (11–25% affected), (3) moderate (26–50% affected), (4) marked (51–75% affected), (5) severe (76–100% affected).

Birefringent imaging was conducted to visualize cSiO_2_ particle deposition in the lung. H&E-stained lung tissues were scanned with an Olympus Slideview VS200 virtual slide scanner as described above. Exposure time was set to 75 ms, and focal points were set to extra high. A randomly selected slide from the VEH/CON group was used to calibrate shading correction and white balance before birefringent imaging, and a randomly selected slide from the cSiO_2_/CON group was used to calibrate and re-zero the polarization angle before scanning each experimental group.

### Quantification of IgG AAbs in BALF and plasma

Apoptotic cell (AC)-derived material was generated for solid-phase in an indirect enzyme-linked immunosorbent assay (ELISA) as previously described (Stearns et al. 2016). Briefly, RAW 264.7 murine macrophages were cultured in 100-mm cell culture dishes in RPMI 1640 medium containing 10% fetal bovine serum (FBS) and 1% penicillin-streptomycin (P/S). The cells were harvested by centrifugation at 500 × *g* for 5 min, resuspended to a final density of 1 × 10^7^ cells/ml in serum-deprived RPMI 1640 medium containing 1% P/S, then treated with 1 μM staurosporine (R&D Systems) to induce apoptosis. Cells were placed in a 37 °C incubator (5% CO_2_) for 24 h, then the supernatant was collected, centrifuged at 500 × *g* for 10 min, and frozen in 2 ml aliquots at −20 °C. cSiO_2_-killed cell (SKC)-derived material for ELISA solid-phase was prepared using a previously described protocol (Rajasinghe et al. 2020). RAW 264.7 macrophages were seeded in 100-mm cell culture dishes at a density of 3.2 × 10^5^ cells/ml in RPMI 1640 medium containing 0.25% FBS and 1% P/S. Then, cells were treated with 50 μg/ml cSiO_2_ to induce robust cell death. Cells were placed in a 37 °C incubator (5% CO_2_) for 20 h, then the supernatant was collected, centrifuged at 500 × *g* for 10 min, and frozen in 2 ml aliquots at −20 °C.

Total dsDNA and protein content in AC-derived material and SKC-derived material were quantitated by using a Quant-iT^™^ PicoGreen^®^ dsDNA Assay Kit (Thermo Fisher Scientific) and a Pierce^™^ BCA Protein Assay Kit (Thermo Fisher Scientific), respectively, according to the manufacturer’s instructions. dsDNA was measured using a FilterMax F3 Multimode plate reader (Molecular Devices) set to fluorescence wavelengths of 480/520 nm. Protein was measured using a FilterMax F3 Multimode plate reader (Molecular Devices) set to an absorbance wavelength of 562 nm. Total dsDNA and protein content in AC-derived and SKC-derived material are recorded in Supplementary Table 3.

IgG AAbs to dsDNA, nucleosomes, AC-derived material, and SKC-derived material were measured in BALF and plasma of mice from the 28 days PI cohort as described previously (Stearns et al. 2016). Briefly, 96-well flat-bottom Nunc-Immuno^™^ MaxiSorp microplates (Thermo Fisher Scientific) were first coated with 20 μg/ml poly-L-lysine in PBS (pH 7.4) and incubated overnight at 4 °C. Plates were washed three times with PBS after all incubation steps. After treating the plates with poly-L-lysine, plates were blocked with 300 μl/well blocking buffer (PBS, 2% [w/v] BSA, 0.05% [v/v] Tween 20) for 2.5 h at room temperature. Then, plates were coated with 50 μl/well of AC-derived supernatant, SKC-derived material, 2.5 μg/ml calf thymus dsDNA (Alpha Diagnostic International), or 2.5 μg/ml calf thymus nucleosomes (Arotec Diagnostics) diluted in ELISA dilution buffer (PBS, 0.1% [w/v] BSA, 0.05% [v/v] Tween 20) and incubated for 1 h at room temperature. Following antigen coating, 50 μl of BALF or plasma diluted 1:20 in ELISA dilution buffer was added to plates and incubated for 1 h at room temperature. Mouse anti-dsDNA antibody (EMD Millipore Corporation, Temecula, CA, USA) was used to establish a standard curve ranging from 2000 arbitrary units (U) to 3.91 U in 2-fold increments. Plates were then incubated with 50 μl/well goat anti-human IgG Fc HRP-conjugated detection antibody (Southern Biotech, Birmingham, AL, USA) diluted 1:5000 in ELISA dilution buffer for 1 h at room temperature. Finally, plates were incubated with 50 μl/well K-Blue^®^ Advanced Plus TMB Substrate (Neogen) for 20 min at room temperature, and sample absorbances measured using a FilterMax F3 Multimode plate reader (Molecular Devices) set to 650 nm. Using SoftMax Pro Software (Molecular Devices), sample absorbances were converted to IgG AAb concentrations (in U/ml) based on the anti-dsDNA antibody standard curve.

### Data analysis and statistics

MetaboAnalyst 6.0 (https://www.metaboanalyst.ca/) (Pang et al. 2024) was used to conduct statistical analyses for LC-MS/MS data as previously described (Favor et al. 2023). Briefly, data were inputted into the one factor statistical analysis module as a comma separated values (.csv) file with features (i.e. metabolites) organized in rows and samples organized in unpaired columns. Data were cleaned by removing features that were missing >70% data and replacing remaining missing values with the limit of detection (LOD; 1/5 of the minimum positive value for each variable) corresponding to the feature in question. Then, auto scaling only was used to normalize the data, and experimental groups were selected for comparisons using the data editor option. One-way analysis of variance (ANOVA) (FDR = 0.05) followed by Tukey’s honestly significant difference (HSD) *post-hoc* test was used to compare experimental groups, with FDR *q* < 0.05 considered statistically significant.

GraphPad Prism Version 9 (GraphPad Software, San Diego, CA, www.graphpad.com) was used to conduct statistical analyses for all other data. The ROUT outlier test (*Q=*1%) and the Shapiro-Wilk test (*p* < 0.01) were used to identify outliers and assess data normality, respectively. For comparisons between the VEH/CON, cSiO_2_/CON, and cSiO_2_/TPPU groups in both 7 days PI and 28 days PI cohorts, non-normal and semi-quantitative data were analyzed by the Kruskal-Wallis nonparametric test followed by Dunn’s *post-hoc* test. The Brown-Forsythe test (*p* < 0.01) was used to test the assumption of equal variances across treatment groups. Normal data with unequal variances were analyzed using the Brown-Forsythe/Welch analysis of variance (ANOVA) test followed by Dunnett’s T3 *post-hoc* test. Normal data that met the assumption of equal variance were analyzed by standard one-way ANOVA followed by Tukey’s *post-hoc* test. For timepoint comparisons within the VEH/CON, cSiO_2_/CON, and cSiO_2_/TPPU groups, non-normal and semiquantitative data were analyzed by the Mann-Whitney nonparametric test. The *F* test (*p* < 0.05) was used to test the assumption of equal variances across the 7 days PI and 28 days PI groups. Normal data with unequal variances were analyzed using an unpaired *t*-test with Welch’s correction. Normal data that met the assumption of equal variance were analyzed using an unpaired *t*-test. Data are presented as mean ± standard error of the mean (SEM), with *p* < 0.05 considered statistically significant.

## Results

### Dietary TPPU distributes in plasma and lung and elicits epoxide metabolite shift in plasma

**Figure 2.**
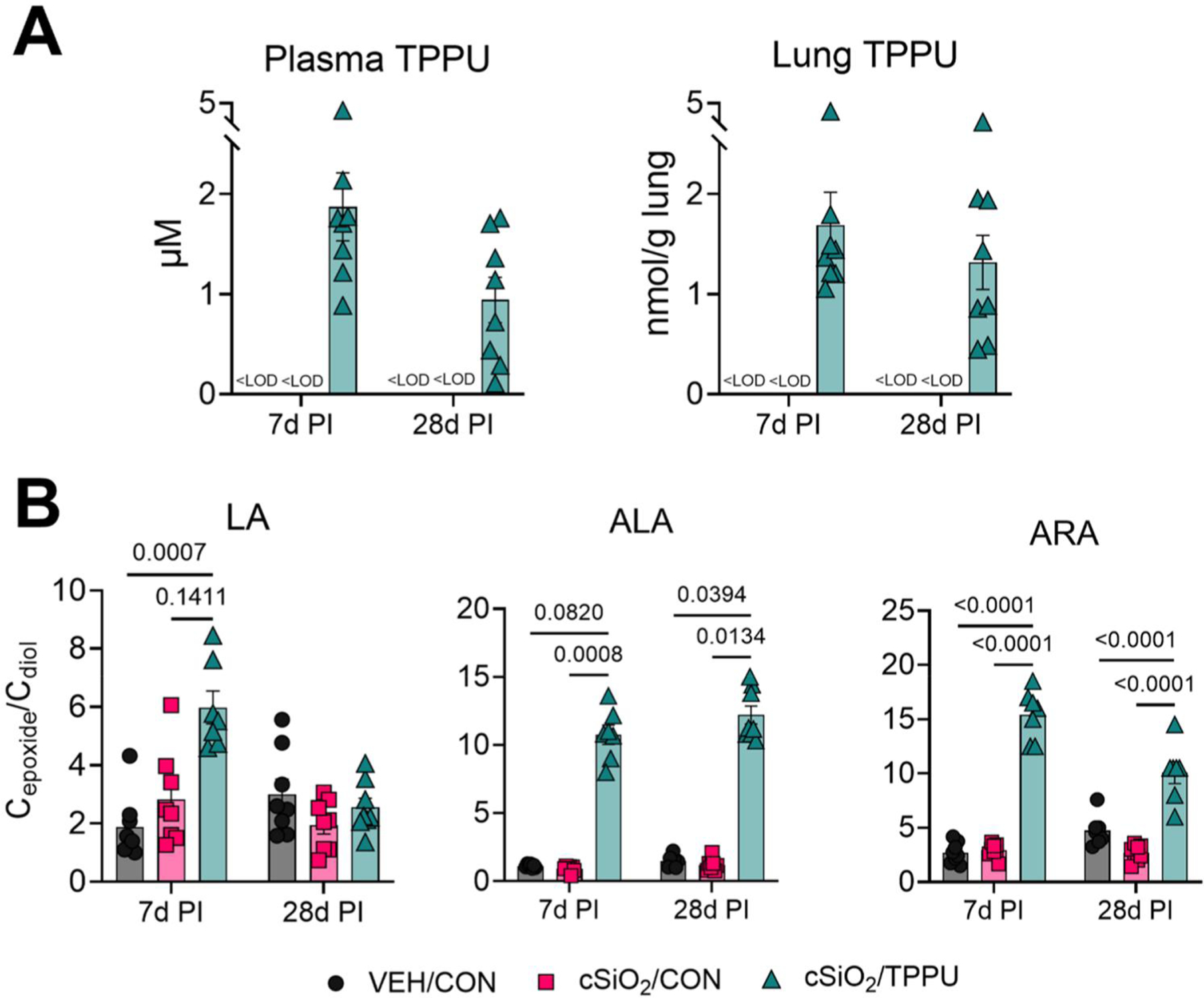
Dietary TPPU supplementation increases drug tissue concentrations and sEH metabolites in plasma. (A) Concentrations of TPPU in lung (presented as nmol/g lung tissue) and plasma (presented as μM) were measured at 7 and 28 days post-cSiO_2_ instillation by LC-MS/MS. Administering TPPU in AIN-93G rodent diet results in elevated TPPU levels in lung tissue and plasma. Data are presented as mean ± SEM (*n=*8). (B) In plasma but not in the lung, TPPU consumption significantly alters epoxide/diol ratios for metabolites derived from LA, ALA, and ARA in plasma. Ratios between sums of all EpFAs and sums of all DiHFAs from each PUFA precursor are shown. Data are presented as mean ± SEM (*n=*8). <LOD; below limit of detection. Values of *p* < 0.2 are shown, with *p* < 0.05 considered statistically significant.

Whole body, kidney, spleen, or liver weights at 7 days PI and 28 days PI were not affected by TPPU or cSiO_2_ treatment (Supplementary Figures 1(A,B)). At 7 days PI and 28 days PI, TPPU concentrations in the plasma were 1.9 and 1 μM, respectively, and in the lung were 1.7 nmol/g and 1.3 nmol/g lung tissue, respectively (Figure 2(A)). At both timepoints, TPPU significantly increased epoxide/diol ratios in plasma for metabolites biosynthesized from ω–3 α-linolenic acid (ALA) and ω–6 arachidonic acid (ARA), compared to the corresponding cSiO_2_/CON groups (Figure 2(B)). Plasma epoxide/diol ratios for metabolites derived from ω–6 linoleic acid (LA) increased non-significantly at 7 days PI and did not change at 28 days PI in mice administered TPPU. In the lung, epoxide/diol ratios for ALA-derived metabolites were significantly elevated by TPPU at 28 days PI, but not for ALA-derived metabolites at 7 days PI nor for LA-and ARA-derived metabolites at both timepoints (Supplementary Figure 2). Together, these findings suggest TPPU was not toxic at the dietary concentration used and effectively distributed to plasma at pharmacologically active levels.

### cSiO_2_-induced production of prostaglandins in lung is not affected by TPPU

**Figure 3.**
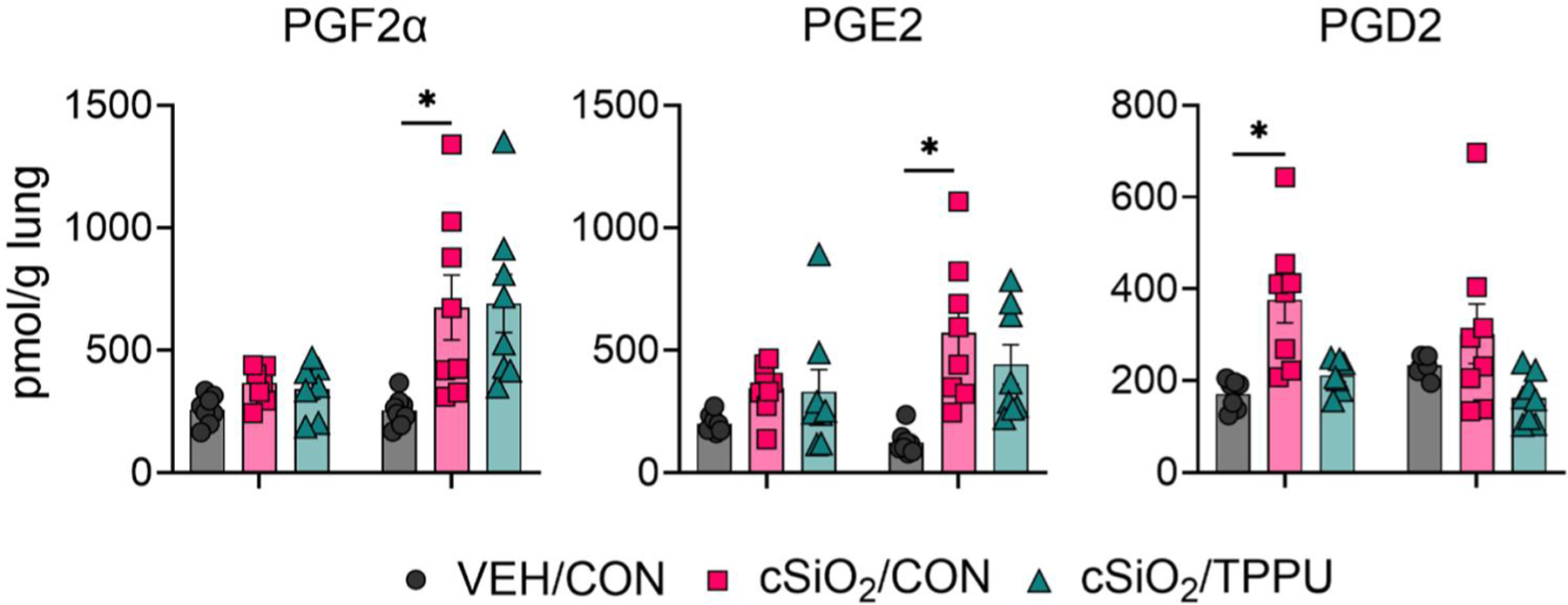
cSiO_2_ induced prostaglandin production in the lungs is modestly affected by TPPU. Following sacrifice, lung tissues were snap frozen, treated with antioxidant cocktail, and homogenates analyzed for production of selected arachidonic acid (ARA)-derived prostaglandin metabolites (PGF2α, PGE2, PGD2) by LC-MS/MS. Metabolite quantities were normalized to the original weight of lung tissue homogenized for the analysis. Data are presented as mean ± SEM (*n=*8). MetaboAnalyst Version 6.0 was used for data normalization and statistical analysis by one-way analysis of variance (ANOVA) (FDR = 0.05) followed by Tukey’s honestly significant difference (HSD) *post-hoc* test. *FDR *q* < 0.05.

At 28 days post-cSiO_2_ instillation, lung levels of ARA-derived prostaglandin F2α (PGF2α) and prostaglandin E2 (PGE2) were elevated compared to time-matched VEH/CON mice (Figure 3). In similar manner, lung levels of prostaglandin D2 (PGD2) were higher in the lungs of cSiO_2_/CON mice at 7 days PI compared to time-matched VEH/CON mice. TPPU did not significantly impact PGF2α, PGE2, or PGD2 levels in the lung at either timepoint.

### cSiO_2_-triggered proinflammatory cytokine, chemokine, and type I IFN gene expression in lung is refractory to TPPU

**Figure 4.**
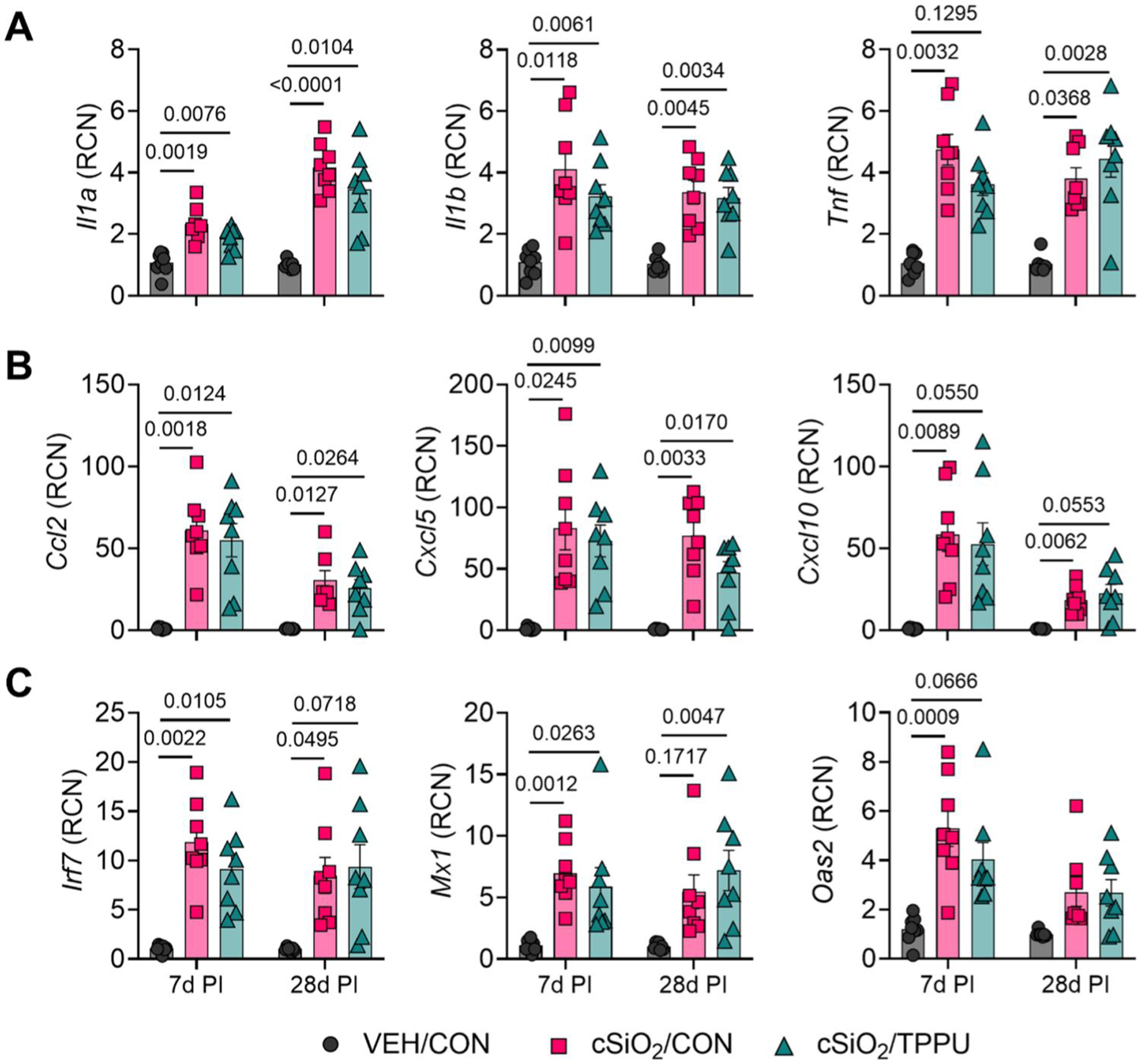
cSiO_2_-induced proinflammatory cytokine, chemokine, and IFN-regulated gene expression in the lung is not affected by TPPU. Following sacrifice, caudal lung lobes were isolated and analyzed for RNA expression of selected (A) proinflammatory cytokines (i.e. *Il1a*, *Il1b*, *Tnf*), (B) chemokines (i.e. *Ccl2*, *Cxcl5*, *Cxcl10*), and (C) type I interferon-regulated genes (i.e. *Irf7*, *Mx1*, *Oas2*). Data are presented as mean ± SEM (*n=*8). Values of *p* < 0.1 are shown, with *p* < 0.05 considered statistically significant.

At both 7 days PI and 28 days PI, cSiO_2_ significantly upregulated expression of selected proinflammatory cytokine (i.e. *Il1a*, *Il1b*, *Tnf*), chemokine (i.e. *Ccl2*, *Cxcl5*, *Cxcl10*), and type I IFN-regulated genes (i.e. *Irf7*, *Mx1*, *Oas2*) in the lung (Figure 4). While mRNA transcript levels for most genes were largely comparable between 7 days PI and 28 days PI, cSiO_2_-exposed mice exhibited higher expression levels for *Ccl2*, *Cxcl10*, and *Oas2* at 7 days PI and *Il1a* at 28 days PI. TPPU did not significantly affect cSiO_2_-induced expression of proinflammatory cytokines, chemokines, and type I IFN-regulated genes.

### cSiO_2_-induced profibrotic cytokine and chemokine protein responses in lung are unaffected by TPPU

**Figure 5.**
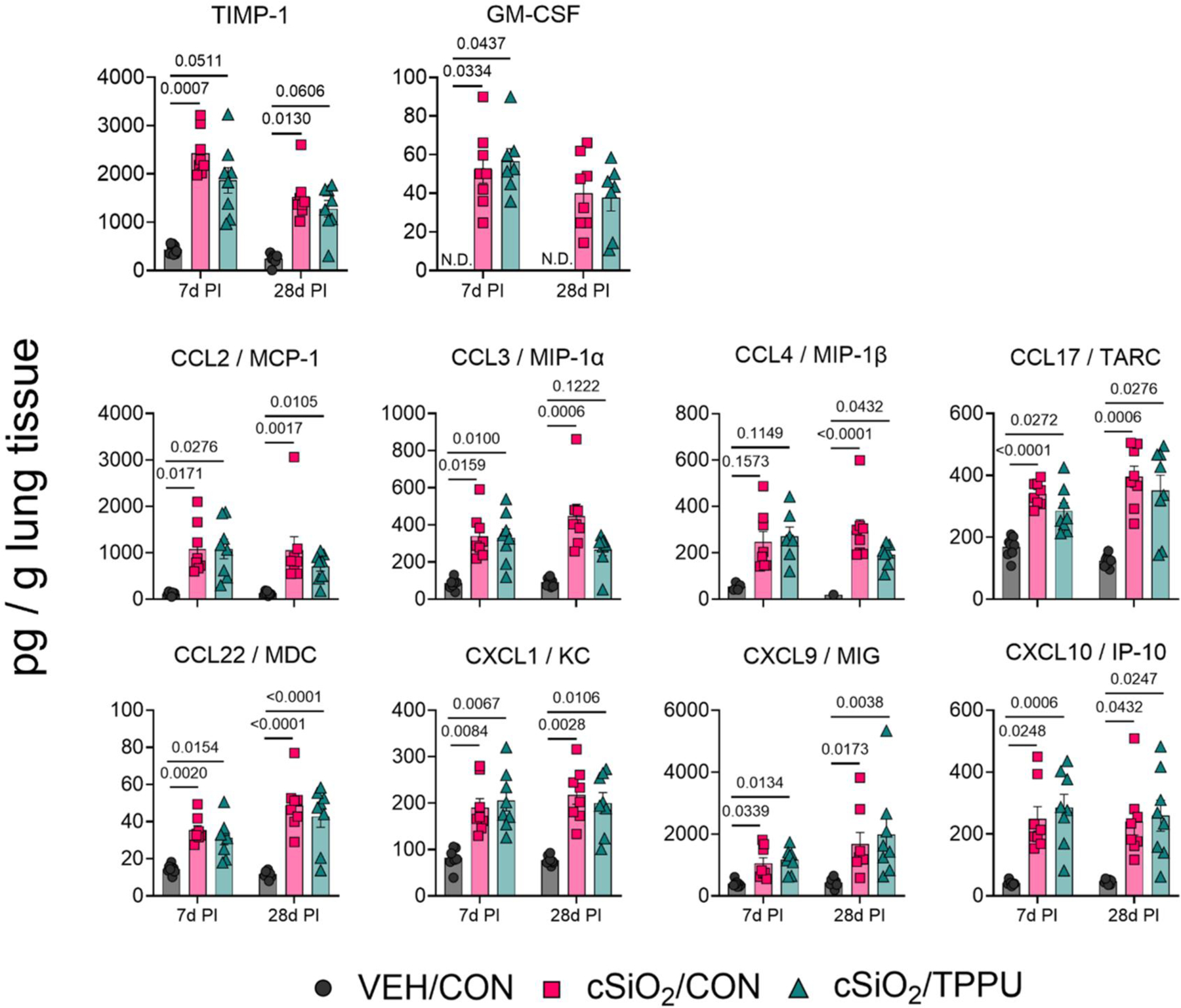
cSiO_2_ induces production of fibrosis-related cytokines and chemokines in the lung. Following sacrifice, middle lung lobes were isolated and homogenates analyzed for production of selected fibrosis-related cytokines (i.e. TIMP-1, GM-CSF), profibrotic chemokines (i.e. MCP-1, MIP-1α, MIP-1β, TARC, MDC, KC), and antifibrotic chemokines (i.e. MIG, IP-10). Protein quantities were normalized to the original weight of lung tissue homogenized for the analysis. Data are presented as mean ± SEM (*n*=8). Values of *p* < 0.2 are shown, with *p* < 0.05 considered statistically significant.

cSiO_2_ triggered robust production of proinflammatory and profibrotic protein mediators in the lung at both 7 days PI and 28 days PI (Supplementary Table 4). Notably, cytokines associated with pulmonary fibrosis (i.e. TIMP-1, GM-CSF) (Isabelle et al. 2012; Zelko et al. 2016), profibrotic chemokines (i.e. MCP-1, MIP-1α, MIP-1β, TARC, MDC, KC), and anti-fibrotic chemokines (i.e. MIG, IP-10) were upregulated by cSiO_2_ exposure at both timepoints (Figure 5). The impacts of cSiO_2_ on other proinflammatory cytokines and T cell-derived cytokines were more limited, as there were only significant increases in TNF-α and IL-16 at 28 days PI, in IL-17 at both timepoints, and in IL-6 at neither timepoint (Supplementary Figure 3). Like gene expression in the lung, TPPU minimally impacted cSiO_2_-induced production of proinflammatory and profibrotic proteins (Figures 4 and 5).

### TPPU suppresses cSiO_2_-induced elevation of monocytes, neutrophils, and lymphocytes in the BALF

**Figure 6.**
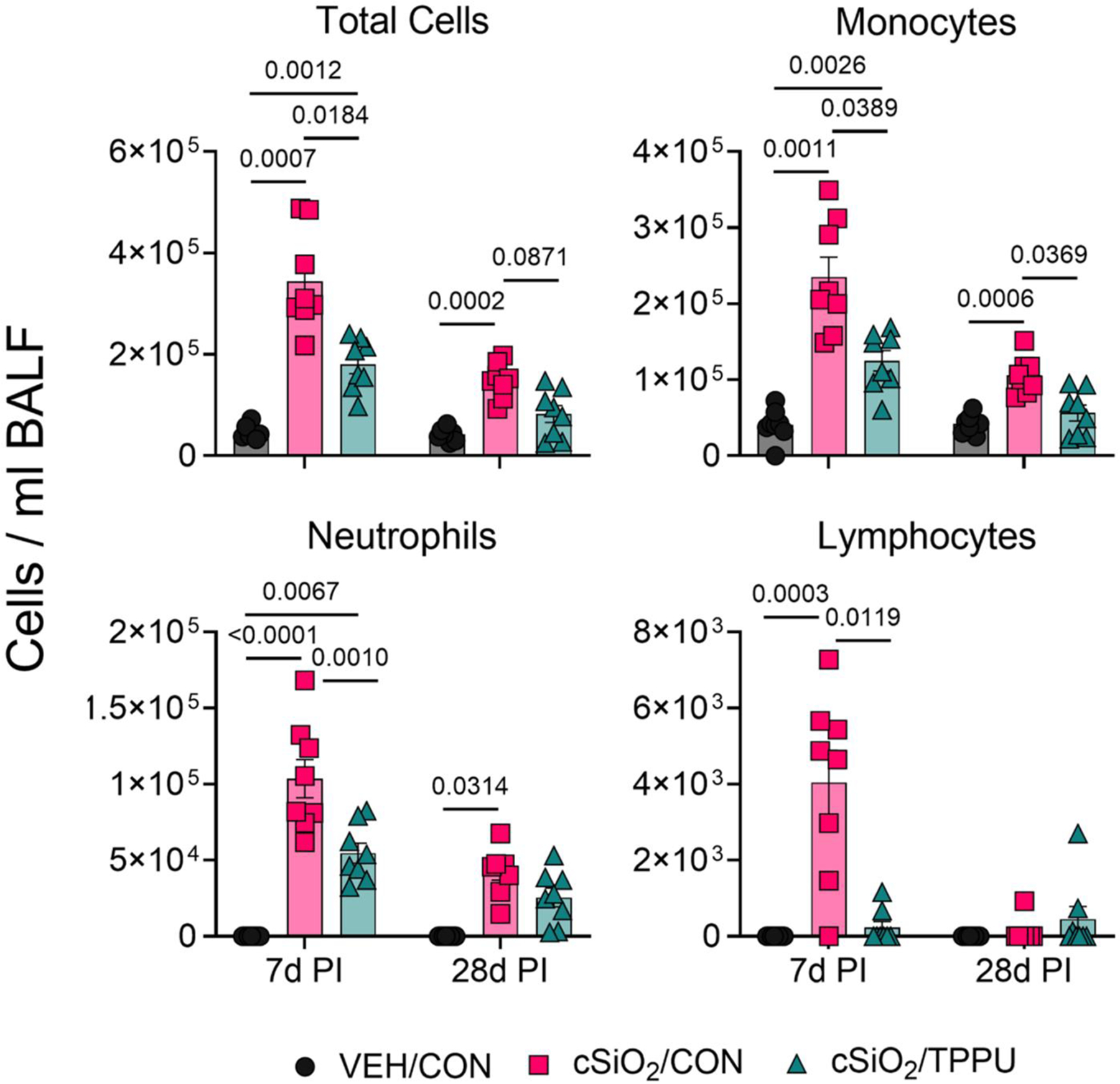
cSiO_2_-induced immune cell accumulation in BALF is suppressed by TPPU. At necropsy, total cells, monocytes, neutrophils, and lymphocytes were quantified in BALF. Data are presented as mean ± SEM. Values of *p* < 0.1 are shown, with *p* < 0.05 considered statistically significant.

Total cells, monocytes, and neutrophils in the BALF of cSiO_2_/CON mice were significantly elevated compared to VEH/CON mice at both 7 days PI and 28 days PI (Figure 6). cSiO_2_/CON mice in the 7 days PI cohort demonstrated markedly higher numbers of total BALF cells, monocytes, and neutrophils compared to cSiO_2_/CON mice in the 28 days PI cohort. Lymphocyte accumulation in the BALF was evident in cSiO_2_/CON mice at 7 days PI but negligible at 28 days PI. TPPU inhibited cSiO_2_-induced increases in total cells, monocytes, neutrophils, and lymphocytes.

### cSiO_2_-triggered inflammatory cell infiltration and fibrosis are modestly impacted by TPPU

**Figure 7.**
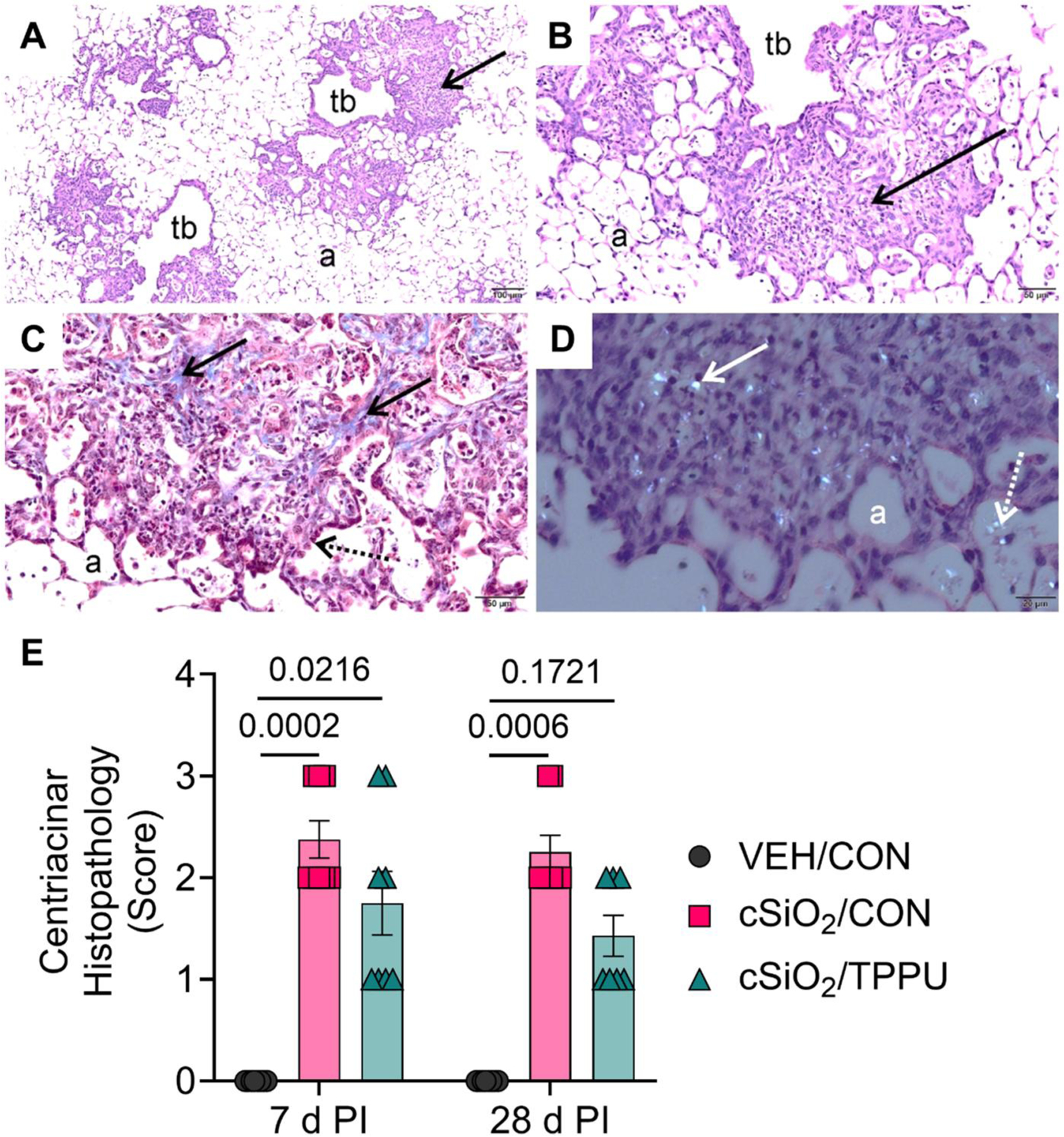
cSiO_2_-induced centriacinar histopathology in the lung is modestly suppressed by TPPU. Light photomicrographs of hematoxylin & eosin (H&E) lung tissues at (A) low and (B) high magnification illustrating chronic centriacinar lesions (solid black arrows) composed of interstitial fibrosis, mixed inflammatory cell inflammation, and alveolar epithelial hyperplasia from cSiO_2_/CON mice sacrificed at 7 days PI. (C) Centriacinar lung lesion (7 days PI) stained with Masson’s trichrome illustrating areas of interstitial fibrosis (blue stain; solid black arrows). (D) H&E-stained centriacinar lung lesion taken with polarized light exposing birefringent cSiO_2_ particles embedded in the fibrotic lesion (solid white arrow) and associated with degenerating and necrotic phagocytic cells in alveolar airspaces (stippled white arrows). (E) Graphical figure of semi-quantitative severity scores for centriacinar histopathology. Scoring was as follows: 0—no significant finding, 1—minimal, 2—mild, 3—moderate, 4—marked, 5—severe. See text for detailed criteria used in severity scoring. Data are presented as mean ± SEM (*n=*8). Values of *p* < 0.2 are shown, with *p* < 0.05 considered statistically significant. a: alveolar parenchyma; tb: terminal bronchiole.

**Figure 8.**
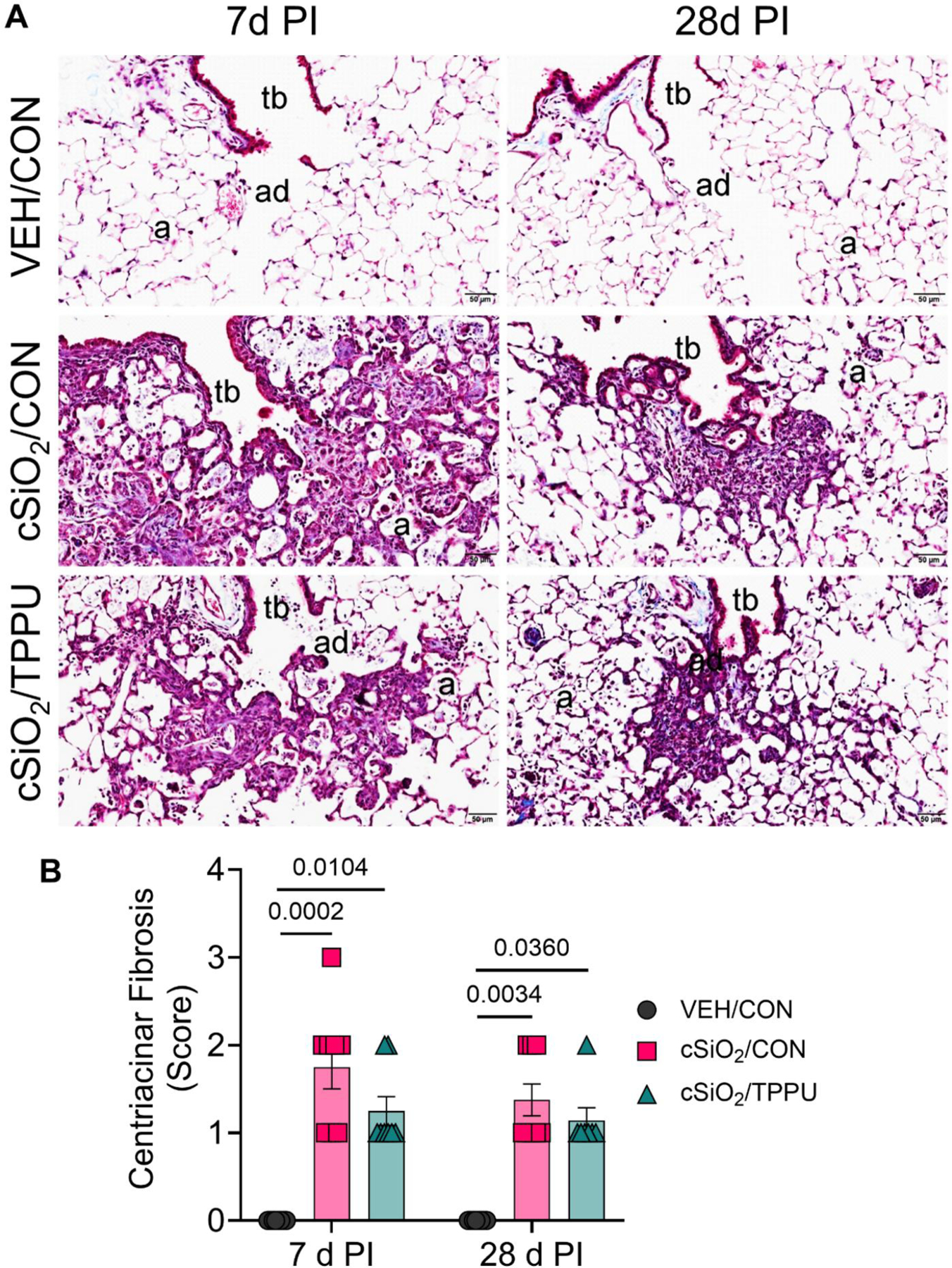
cSiO_2_-induced centriacinar fibrosis in the lung is not significantly impacted by TPPU. (A) Representative light photomicrographs of Masson’s trichrome-stained lung tissues (centriacinar regions) from VEH/CON, cSiO_2_/CON, and cSiO_2_/TPPU mice sacrificed at 7 days PI and 28 days PI. (B) Semi-quantitative severity scores for centriacinar interstitial fibrosis. Scoring was as follows: 0—no significant finding, 1—minimal, 2—mild, 3—moderate, 4—marked, 5—severe. See text for detailed criteria used in severity scoring. Data are presented as mean ± SEM (*n=*8). Values of *p* < 0.1 are shown, with *p* < 0.05 considered statistically significant. a: alveolar parenchyma; ad: alveolar duct; tb: terminal bronchiole.

**Figure 9.**
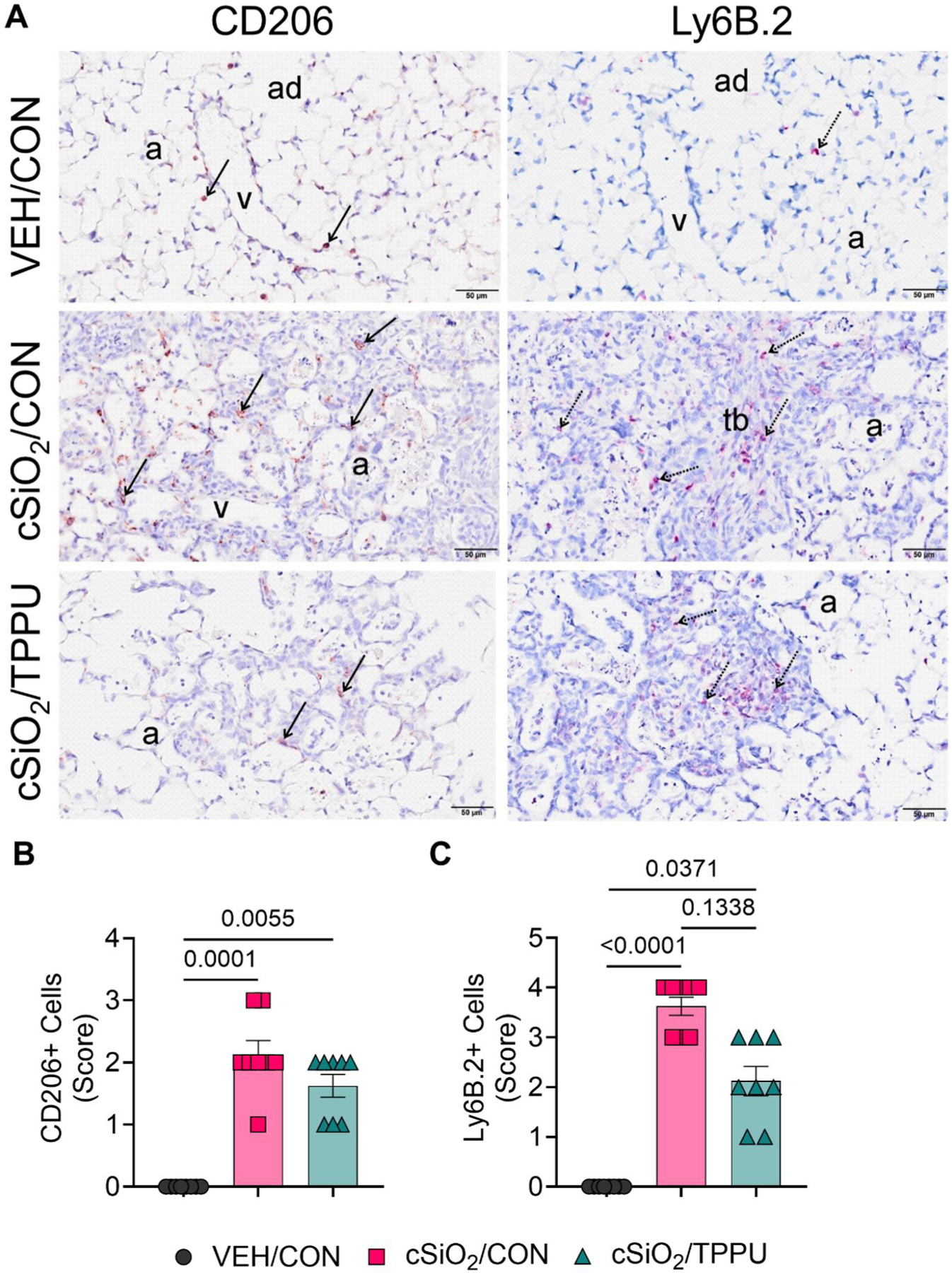
cSiO_2_-induced infiltration of CD206^+^ alveolar macrophages/monocytes and Ly6B.2^+^ neutrophils in the lung is modestly affected by TPPU. (A) Representative light photomicrographs of lung tissues (centriacinar regions) from VEH/CON, cSiO_2_/CON, and cSiO_2_/TPPU mice sacrificed at 7 days PI. Lung tissues were immunohistochemically stained for CD206^+^ alveolar macrophages/monocytes (brown chromagen) and Ly6B.2^+^ neutrophils (red chromagen). Semi-quantitative scores for the presence of (B) CD206^+^ cells and (C) Ly6B.2^+^ cells in the centriacinar regions of the lung. Severity scores were as follows: 0—no significant finding, 1—minimal, 2—mild, 3—moderate, 4—marked, 5—severe. See text for detailed criteria used in severity scoring. Data are presented as mean ± SEM (*n=*8). Values of *p* < 0.2 are shown, with *p* < 0.05 considered statistically significant. a: alveolar parenchyma; ad: alveolar duct; tb: terminal bronchiole; v: pulmonary vein. Solid arrow, CD206+ cell; stippled arrow, Ly6B.2+ cell.

CON-fed mice instilled with VEH had no lung histopathology at either 7 days PI or 28 days PI. In contrast, cSiO_2_-instilled CON-fed mice had multifocal, fibrotic, and proliferative lung lesions in centriacinar regions of the lung, primarily in the proximal alveolar ducts (Figures 7 and 8). These focal lesions were composed of intramural interstitial fibrosis, hyperplasia of alveolar epithelial type 2 and transitional cells, and a mixed inflammatory cell infiltration (alveolitis) composed primarily of CD206^+^ macrophages/monocytes and Ly6B.2^+^ neutrophils (Figure 9).

Numerous widely scattered birefringent cSiO_2_ particles were embedded in the thickened centriacinar interstitial tissue and in associated alveolar airspaces that contained proteinaceous material and cellular debris resulting from degenerating or necrotic phagocytic macrophages (Figures 7(D) and 9(A)). Lesser amounts of cSiO_2_ particles, inflammatory cells, macrophages/monocytes, and cellular debris were present in airspaces of distal alveolar regions of the lung that were also without the hyperplasia of alveolar type 2 or transitional epithelial cells and septal fibrosis found in the more proximal centriacinar areas.

Overall, mice fed TPPU trended toward less cSiO_2_-induced fibrosis and CD206^+^ macrophage/monocyte and Ly6B.2^+^ neutrophil infiltration but these differences did not reach statistical significance (Figures 7–9).

### cSiO_2_-induced ELS development and AAb production in not affected by TPPU

**Figure 10.**
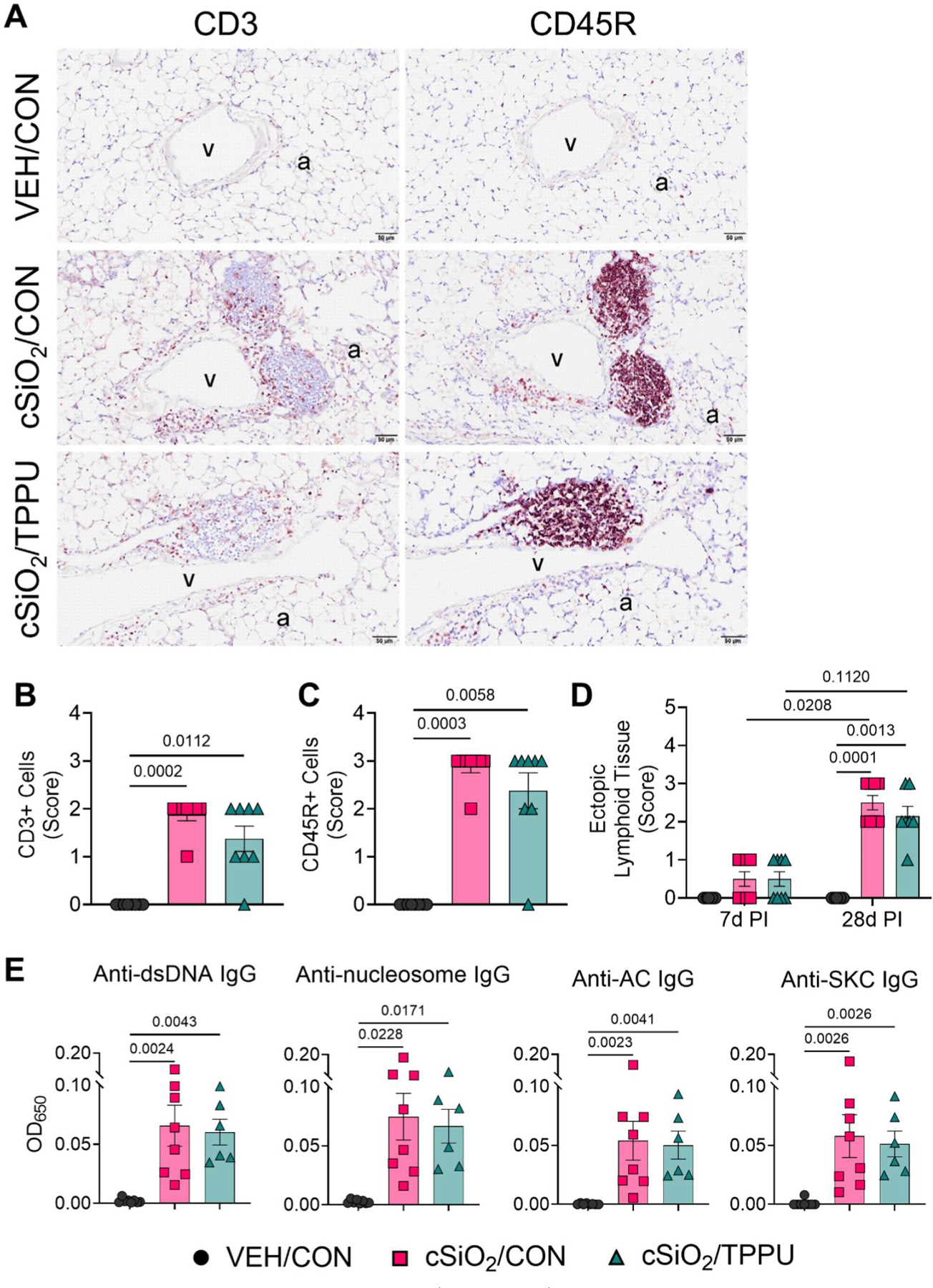
cSiO_2_-induced perivascular and peribronchiolar infiltration of CD3^+^ and CD45R^+^ lymphocytes in the lung are not affected by TPPU. (A) Representative light photomicrographs of lung tissues from VEH/CON, cSiO_2_/CON, and cSiO_2_/TPPU mice sacrificed at 28 days PI. Lung tissues were immunohistochemically labeled for CD3^+^ T lymphocytes and CD45R^+^ B lymphocytes (brown chromagen). Semi-quantitative severity scores for the presence of (B) CD3^+^ cells, (C) CD45R^+^ cells, and (D) development of ectopic lymphoid tissue in perivascular and peribronchiolar interstitial tissue. Severity scores for CD3^+^ cells and CD45R^+^ cells were identical. Severity scores were as follows: 0—no significant finding, 1—minimal, 2—mild, 3—moderate, 4—marked, 5—severe. See text for detailed criteria used in severity scoring. Data are presented as mean ± SEM (*n=*8). Values of *p* < 0.2 are shown, with *p* < 0.05 considered statistically significant. a: alveolar parenchyma; v: pulmonary vein. (E) Total IgG specific to dsDNA, nucleosome antigen, apoptotic cell (AC)-derived material, and cSiO_2_-killed cell (SKC)-derived material was measured by ELISA in the BALF and plasma of VEH/CON, cSiO_2_/CON, and cSiO_2_/TPPU mice sacrificed at 28 days PI. Data are presented as mean ± SEM (*n=*8). Values of *p* < 0.2 are shown, with *p* < 0.05 considered statistically significant.

Conspicuous accumulation of CD3^+^ and CD45R^+^ lymphoid cells, indicative of T and B cells, respectively, and elevated ELS scores in perivascular and peribronchiolar interstitial tissue were evident in the lungs of cSiO_2_-treated mice after 28 days PI (Figures 10(A–D)). Corresponding with increased ELS scores, mice that received cSiO_2_ displayed significant elevations in IgG AAbs specific to dsDNA, nucleosome antigen, AC-derived material, and SKC-derived material in the BALF (Figure 10(E)). In the plasma, cSiO_2_ triggered modest, yet insignificant, increases in IgG specific to dsDNA, nucleosome antigen, SKC-derived material, and AC-derived material (data not shown). TPPU administration did not significantly influence cSiO_2_-induced B and T cell accumulation, ELS development, or AAb production (Figures 10(A–E)).

## Discussion

Environmental exposure to respirable cSiO_2_ has been etiologically linked to the development of pulmonary fibrosis and systemic autoimmune disease in mice and humans (Brown et al. 2003; Re et al. 2014; Wollin et al. 2014; Zhao et al. 2020). This investigation is the first to assess the efficacy of the sEH inhibitor TPPU, a well-established lipidome-modifying agent, against acute cSiO_2_-triggered lung inflammation, pulmonary fibrosis, and early autoimmunity in lupus-prone mice. It was found that a single IN dose of cSiO_2_ induces (i) leukocyte accumulation in the BALF, (ii) centriacinar inflammation, centriacinar fibrosis, and perivascular ELS development, (iii) monocyte and neutrophil recruitment, (iv) accumulation of CD3^+^ T lymphocytes and CD45R^+^ B lymphocytes in ELT, (v) expression of proinflammatory cytokine, chemokine, and type I IFN-regulated mRNAs and proteins, and (vi) secretion of AAb targeting dsDNA, nucleosomes, apoptotic cell AAg, and cSiO_2_-killed cell AAg in alveolar fluid. Importantly, it was further determined that while TPPU significantly decreased differential immune cell counts in the BALF and modestly reduced CD206^+^ monocytes and Ly6B.2^+^ neutrophils in the lung, this drug’s effects on other measured endpoints were negligible.

Pulmonary collagen deposition was first observed at 7 days PI, followed by ELS neogenesis at 28 days PI, indicating that fibrosis may precede and contribute to lymphoid tissue development. Perera et al. (2023) found similar ELS formation in sheep exposed to bleomycin (BLM), and human studies of IPF have shown CD20^+^ B cell aggregates and other immune markers in fibrotic lungs (Todd et al. 2013; Xue et al. 2013; Hoyne et al. 2017). This suggests that T and B lymphocytes in ELS may exacerbate fibrosis, although direct causality remains unexplored. Further research is needed to assess the influence of cSiO_2_-induced ELS on collagen deposition and fibroblast behavior.

At 28 days PI, cSiO_2_-instilled mice exhibited elevated lung PGF2α and PGE2, both of which influence fibrosis. PGE2 typically inhibits fibrosis by acting on the EP2 and EP4 receptors in fibroblasts (Breyer et al. 2001; Garrison et al. 2013; Sieber et al. 2018; Li et al. 2021; Teegala et al. 2023), while PGF2α promotes fibrosis through the FP receptor (Breyer et al. 2001; Oga et al. 2009; Li et al. 2021) and PGE2 by binding to the EP1/EP3 receptors (Breyer et al. 2001; Chen et al. 2015; Guo et al. 2020; Li et al. 2021). PGD2, which was elevated at 7 days PI, appears to play an anti-fibrotic role by inhibiting inflammation and fibroblast activity (Ayabe et al. 2013; Kida et al. 2016; Ueda et al. 2019). This suggests that cSiO_2_ may elicit both pro- and anti-fibrotic prostaglandin responses. Further studies should examine how these prostaglandins interact with their receptors in lung fibroblasts and whether they contribute to ELS formation.

Increased TIMP-1 levels in the lungs of cSiO_2_/CON mice at both timepoints suggest it may contribute to fibrosis by inhibiting matrix metalloproteinases that degrade ECM proteins like collagen (Selman et al. 2000; Madtes et al. 2001; Dong and Ma 2017; Jackson et al. 2017; Cabral-Pacheco et al. 2020). GM-CSF, which was also elevated, has dual roles: it can both reduce fibrosis by enhancing PGE2 production and promote it through TGF-β and myofibroblast accumulation (Xing et al. 1997; Moore et al. 2000; Joshi et al. 2020). These findings imply that the role of GM-CSF in cSiO_2_-induced fibrosis is complex and warrants further investigation. cSiO_2_ also induced the production of several chemokines associated with fibrosis (MCP-1, MIP-1α, MIP-1β, TARC, MDC, KC), which promote inflammation, fibroblast activation, and ECM remodeling (Driscoll 1994; Smith et al. 1994, Smith et al. 1995; Hogaboam et al. 1999; Belperio et al. 2004; Inoue et al. 2004; Strieter et al. 2007; Yogo et al. 2009; Liu et al. 2015; Cheng et al. 2019; Wang et al. 2023). Additionally, MIG and IP-10, which are involved in vascular remodeling, were upregulated, though these chemokines are typically angiostatic and play protective roles in other fibrotic conditions (Keane et al. 1999; Antoniou et al. 2006; Strieter et al. 2007). This highlights the need for further research on how these chemokines contribute to fibrosis and vascular remodeling in cSiO_2_ exposure.

In female lupus-prone NZBWF1 mice, we have previously demonstrated that a single IN dose of 2.5 mg cSiO_2_ triggers robust lung inflammation and upregulated chemokines and IFN-regulated genes (Chauhan et al. 2021). Consistent with the prior study, herein we observed increased neutrophils and monocytes in BALF, moderate centriacinar inflammation, and upregulation of proinflammatory chemokine and type I IFN mRNA transcripts in the lung. Although BALF leukocyte counts decreased by 28 days PI, the persistence of cSiO_2_ likely sustained a cycle of neutrophilic and monocytic infiltration, tissue damage, and exacerbated inflammatory responses in both studies.

We previously reported in our acute model of cSiO_2_-induced lung toxicity that lipidomic modulation with ω–3 PUFAs suppresses cSiO_2_-triggered cell death in the lung at 7 days PI, total cell and lymphocyte recruitment in the BALF at 28 days PI, autoimmune gene transcription in the lung at 28 days PI, and AAb secretion in the lung at 28 days PI (Chauhan et al. 2024). We posited here that lipidomic modulation with the sEH inhibitor TPPU would also improve biomarkers of lung inflammation and fibrosis following acute cSiO_2_ exposure. While TPPU suppressed immune cell accumulation in the alveolar fluid and centriacinar lung tissue, TPPU did not ameliorate cSiO_2_-induced centriacinar fibrosis in the lung, in contrast to previously published studies. For example, Zhou et al. reported that sEH inhibition by TPPU significantly reduced BLM-induced collagen deposition in the lung at 14 and 21 days PI, as well as TGF-β1-induced activation and differentiation of mouse fibroblasts *in vitro* without eliciting notable toxicity (Zhou et al. 2016). In addition, TPPU pretreatment of primary human lung fibroblasts from IPF patients significantly dampened TGF-β1-mediated fibroblast activation by suppressing expression of type I collagen (Kim et al. 2021). It is possible that the cSiO_2_ dose we used evoked fibrosis to a greater degree of severity than the studies described above, which may explain why the therapeutic effects of TPPU were minimal in our model. Therefore, it would be informative to repeat the experiment described here with smaller cSiO_2_ doses to verify the effectiveness of sEH inhibition against cSiO_2_-triggered lung inflammation and fibrosis.

In the present study, TPPU reached levels of 1.9 and 1 μM in the plasma at 7 days PI and 28 days PI, respectively, corresponding to values ~ 800- and 400-fold greater than the Ki of sEH. Previously, we found that feeding female NZBWF1 mice with TPPU-supplemented AIN-93G diet resulted in a drug plasma concentration of ~ 5 μM (Favor et al. 2023), but those mice were administered TPPU for one additional week compared to the present study. Herein, we found that TPPU significantly increased epoxide/diol ratios for LA, ALA, and ARA metabolites in the plasma. In the lung, TPPU levels reached 1.7 and 1.3 nmol/g at 7 days PI and 28 days PI, respectively, which has not yet been reported in mice, yet epoxide/diol ratios were not significantly impacted, suggesting that these drug levels did not result in optimal sEH inhibition. Although we did not observe abnormal patterns of dietary intake during the study, the reduction in lung and plasma TPPU levels between 7 days PI and 28 days PI—and corresponding lack of efficacy on cSiO_2_-induced fibrosis and autoimmunity—might be attributed to increased drug metabolism and excretion between these timepoints. TPPU is primarily metabolized by Phase I oxidation and amide hydrolysis and excreted *via* urine and feces in rats (Wan et al. 2019). Furthermore, another possibility is that cSiO_2_ exposure contributed to increased expression of Phase I metabolizing enzymes in the liver, kidney, and lung, resulting in reduced TPPU levels between 7 days PI and 28 days PI. In future studies, it would be informative to measure TPPU metabolites in NZBWF1 mouse excrement, as well as compare mRNA expression of Phase I metabolizing enzymes in the liver, kidney, and lung between cSiO_2_-instilled and VEH-instilled mice.

One limitation of our study is that we only analyzed the impacts of cSiO_2_ and TPPU on the whole lung and not specific cell types within the lung (e.g. AMs, airway epithelial cells, fibroblasts, etc.). In several previous studies, cSiO_2_ has been shown to elicit lysosomal membrane permeabilization, mitochondrial dysfunction, NLRP3 inflammasome formation, proinflammatory cytokine production, proinflammatory eicosanoid production, and death and impair efferocytosis in primary AMs and AM-like cells (Hamilton et al. 2008; Lescoat et al. 2020; Favor et al. 2023). Moreover, cSiO_2_ induces MAPK phosphorylation (Øvrevik et al. 2004), DNA damage and cytotoxicity (Fanizza et al. 2007; Wu et al. 2020), MAPK- and NF-kB-dependent cyclooxygenase-2 (COX-2) expression (Tomaru and Matsuoka 2011), NLRP3-dependent mitochondrial depolarization (Wu et al. 2020), and overexpression of profibrotic sulfatase-1 (Perkins et al. 2018) in airway epithelial cells. It has also been demonstrated that cSiO_2_ can upregulate COX-2 and PGE synthase activity directly in human lung fibroblasts, resulting in increased PGE2 production (O’Reilly et al. 2005). In a co-culture system, transcription of collagen I was increased in fibroblasts co-cultured with cSiO_2_-exposed AMs (Li et al. 2017), and NLRP3-dependent IL-1β secretion from cSiO_2_-exposed macrophages elicits robust fibroblast differentiation into myofibroblasts (Hindman and Ma 2019). Accordingly, cSiO_2_ elicits a multifaceted inflammatory response involving many different cell types and pathways, so sEH inhibition by itself may not be sufficient to ameliorate this response. Single cell transcriptomics using the whole lung would provide more insight into the profibrotic effects of acute cSiO_2_ exposure and potential therapeutic effects of TPPU in our current model.

Another limitation is that we did not analyze the impacts of TPPU on cSiO_2_-induced toxicity before 7 days PI or between 7 days PI and 28 days PI. While we observed that TPPU did not significantly change expression and production of proinflammatory cytokines and chemokines in the lung at 7 days PI, it is possible that TPPU may exhibit notable therapeutic effects on these endpoints within the first week after cSiO_2_ exposure. For instance, Bettaieb et al. demonstrated in a murine model of acute pancreatitis that sEH inhibition by TPPU significantly downregulated expression of *Il1b*, *Il6*, and *Tnf* in the pancreas, as well as protein levels of IL-1β, IL-6, and TNF-α in the plasma, up to 48 h after induction of pancreatitis (Bettaieb et al. 2015). In addition, we previously found that acute cSiO_2_ exposure caused marked upregulation and secretion of IL-6 from the lung at 1 day PI but not at 7 days PI, and CD3^+^ T cells and CD45R^+^ B cells began infiltrating the lung at 14 days PI (Chauhan et al. 2021). The 7 days PI and 28 days PI timepoints in the present study were selected to highlight the impacts of cSiO_2_ on lung inflammation and early autoimmunity; however, changes in endpoints between the timepoints could not be adequately tracked. In future studies, timepoints before 7 days PI and between 7 days PI and 28 days PI should be considered to better understand initial events underlying cSiO_2_-induced lung toxicity, mechanisms underlying the transition between innate and adaptive immunity, and potential therapeutic effects of sEH inhibition in this model.

## Conclusion

This study is the first to query the short-term effects of a singular cSiO_2_ dose and the sEH inhibitor TPPU on pulmonary fibrosis and ELS neogenesis using female lupusprone NZBWF1 mice. Notably, cSiO_2_ triggered prostaglandin production, gene expression, proinflammatory and profibrotic cytokines/chemokine release, innate and adaptive immune cell infiltration, centriacinar histopathology, collagen deposition, ELS development, and autoantibody secretion in the lung at 7 days PI and 28 days PI. While sEH inhibition by TPPU suppressed the accumulation of proinflammatory neutrophils, monocytes, and lymphocytes in BALF with no perceivable drug-related toxicity, it did not have therapeutic benefits for the other endpoints. Overall, our findings suggest that in future investigations, it will be important to elucidate linkages between cSiO_2_-induced pulmonary fibrosis and ELS neogenesis in the lung to identify potential therapeutic targets for other drugs.

## Supplementary Material

Supp 1

## Figures and Tables

**Table 1.** Experimental diet formulations.

Experimental diet		
	CON	TPPU
	
Macronutrient	(g/kg total diet)	
Carbohydrates		
Corn starch	398	398
Maltodextrin (dyetrose)	132	132
Sucrose	100	100
Cellulose	50	50
kcal (% of total)	63.2	63.2
Proteins		
Casein	200	200
L-cysteine	3	3
kcal (% of total)	19.7	19.7
Fats^a^		
Corn oil^b^	10	10
High oleic-safflower oil^c^	60	60
kcal (% of total)	17.1	17.1
Other		
AIN-93G mineral mix	35	35
AIN-93G vitamin mix	10	10
Choline bitartrate	3	3
TBHQ antioxidant	0.01	0.01
TPPU	0	0.0225

All values are reported as mass (g) per kg of diet.

a As reported by the manufacturer.

b Corn oil contained 612 g/kg linoleic acid and 26 g/kg oleic acid.

c High oleic-safflower oil contained 750 g/kg oleic acid and 140 g/kg linoleic acid.

**Table 2.** Experimental groups.

Experimental group	Number of animals (*n*)	cSiO_2_ (−/+)	Necropsy timepoint	Experimental diet
VEH/CON	8	−	7 days PI	AIN-93G
cSiO_2_/CON	8	+	7 days PI	AIN-93G
cSiO_2_/TPPU	8	+	7 days PI	AIN-93G+TPPU
VEH/CON	8	−	28 days PI	AIN-93G
cSiO_2_/CON	8	+	28 days PI	AIN-93G
cSiO_2_/TPPU	8	+	28 days PI	AIN-93G+TPPU

VEH: vehicle; CON: control; cSiO_2_: crystalline silica; PI: post cSiO_2_-instillation.

## Data Availability

Original protein data derived from the Mouse Cytokine/Chemokine 44-Plex Discovery Assay^®^ Array are available in a publicly accessible repository: https://doi.org/10.5061/dryad.wh70rxwx3.
