# Supplementary material for: Impact of soluble epoxide hydrolase inhibition on silica-induced pulmonary fibrosis, ectopic lymphoid neogenesis, and autoantibody production in lupus-prone mice
Source: Inhal Toxicol. Author manuscript; Available in PMC 2024 Nov 30. (PMC11606782; doi:10.1080/08958378.2024.2413373)
Supplement: Supp 1 [file NIHMS2028121-supplement-Supp_1.docx]

***Supplementary Material***

**Impacts of Soluble Epoxide Hydrolase Inhibition on Silica-Induced Pulmonary Fibrosis, Ectopic Lymphoid Neogenesis, and Autoantibody Production in Lupus-Prone Mice**

Olivia F. McDonald^a,b,c^, James G. Wagner^b,d^, Ryan P. Lewandowski^d^, Lauren K. Heine^a,b,e^, Vanessa Estrada^c^, Elham Pourmand^f^, Megha Singhal^a^, Jack R. Harkema^a,b,d^, Kin Sing Stephen Lee^a,b,f^, James J. Pestka^b,c,g*^

*^a^Department of Pharmacology and Toxicology, College of Osteopathic Medicine, Michigan State University, East Lansing, Michigan, United States of America; ^b^Institute for Integrative Toxicology, Michigan State University, East Lansing, Michigan, United States of America;* *^c^Department of Microbiology, Genetics, and Immunology, Michigan State University, East Lansing, Michigan, United States of America; ^d^Department of Pathobiology and Diagnostic Investigation, Michigan State University, United States of America; ^e^Los Alamos National Laboratory, Los Alamos, New Mexico, United States of America; ^f^Department of Chemistry, Michigan State University, East Lansing, Michigan, United States of America; ^g^Department of Food Science and Human Nutrition, Michigan State University, East Lansing, Michigan, United States of America*

*Corresponding author: Dr. James J. Pestka, Department of Microbiology, Genetics, and Immunology, Michigan State University, 4176 Biomedical Physical Sciences, 567 Wilson Rd., East Lansing, Michigan 48824-1224; Email: pestka@msu.edu

Supplementary Table 1. List of key reagents, chemicals, and kits.

| Reagent | Vendor | Catalog Number |
| --- | --- | --- |
| AIN-93G Purified Rodent Diet without Vitamin Mix | Dyets Inc. | 110700 |
| AIN-93G VX Vitamin Mix | Dyets Inc. | 310025 |
| LouAna Safflower Oil | LouAna Oils |  |
| Mazola Corn Oil | Mazola |  |
| Microalgal Oil Containing 40% DHA | DHASCO |  |
| TPPU | Synthesized in-house |  |
| Crystalline Silica | U.S. Silica | Min-U-Sil-5 |
| Monoclonal Rat Anti-Mouse Ly6B.2 Alloantigen Antibody | BioRad | MCA771G |
| Polyclonal Rabbit Anti-Mouse CD206 Antibody | Abcam | ab64693 |
| Polyclonal Rabbit Anti-Mouse CD3 Antibody | Abcam | ab5690 |
| Monoclonal Rat Anti-Mouse CD45R Antibody | Becton Dickinson | 550286 |
| RNeasy Mini Kit | Qiagen | 74104 |
| Mouse Cytokine/Chemokine 44-Plex Discovery Assay® Array | Eve Technologies | MD44 |
| Nunc 96-Well Flat Bottom MaxiSorp Immuno Plates | Thermo Fisher Scientific | 442404 |
| Poly-L-Lysine Solution, 0.1% (w/v) | Sigma Aldrich | P8920 |
| Purified Native dsDNA, Calf Thymus | Alpha Diagnostic International | DNAD25-N-1 |
| Purified Nucleosome Antigen, Bovine Thymus | Arotec Diagnostics | ATN02 |
| Anti-DNA Antibody, double stranded, clone BV16-13 | Millipore Sigma | MAB030 |
| Staurosporine | R&D Systems | 1285 |
| Goat Anti-Mouse IgG (heavy-chain sp.)-HRP conjugate | Alpha Diagnostic International | 40120 |
| Quant-iT^™^ PicoGreen^®^ dsDNA Assay Kit | Thermo Fisher Scientific | P7589 |
| Pierce™ BCA Protein Assay Kit | Thermo Fisher Scientific | 23225 |
| RNeasy Mini Kit | Qiagen | 74104 |
| RIPA Lysis and Extraction Buffer | Thermo Fisher Scientific | 89901 |

Supplementary Table 2. Waters TQ-XS tandem quadrupole UPLC/MS/MS linear gradient chromatographic method for analyte separation.

| Mobile phase | A: 0.1% acetic acid in water | B: 84:16 acetonitrile/methanol + 0.1% acetic acid |
| --- | --- | --- |
| Gradient (minutes) | Percentage | Percentage |
| Initial | 65.0 | 35.0 |
| 1.00 | 60.0 | 40.0 |
| 3.00 | 45.0 | 55.0 |
| 8.50 | 35.0 | 65.0 |
| 12.50 | 28.0 | 72.0 |
| 15.00 | 18.0 | 82.0 |
| 16.00 | 0.0 | 100.0 |
| 18.10 | 65.0 | 35.0 |

Injection Volume: 10 µl

Flow Rate: 250 µl/min

Supplementary Table 3. Total dsDNA and protein content in AC-derived material and SKC-derived material generated for AAb ELISAs.

|  | AC-derived material | SKC-derived material |
| --- | --- | --- |
| Total dsDNA (ng/ml) | 2390 | 375 |
| Total protein (µg/ml) | 1381 | 737 |
| ng dsDNA/mg protein | 1731 | 508 |
| dsDNA used in ELISAs (ng/ml) | 10 | 10 |
| Protein used in ELISAs (µg/ml) | 20 | 6 |

Supplementary Table 4. Lung homogenate cytokine levels as determined by Eve Technologies Mouse Cytokine/Chemokine 44-Plex Discovery Assay® Array

|  | VEH/CON  7d PI | cSiO_2_/CON  7d PI | cSiO_2_/TPPU  7d PI | VEH/CON  28d PI | cSiO_2_/CON  28d PI | cSiO­_2_/TPPU  28d PI |
| --- | --- | --- | --- | --- | --- | --- |
| Cytokine | *(pg/g lung tissue, mean ± SEM)* | | | | | |
| CCL2/MCP-1 | 121.3±13.3^A^ | 1080±191.4^B^ | 1084±210.6^B^ | 124.3±14.3^a^ | 1054±293.9^b^ | 710.6±106.5^b^ |
| CCL3/MIP-1α | 85.38±9.58^A^ | 339.8±48.9^B^ | 331.0±48.1^B^ | 92.99±7.79^a^ | 446.2±65.2^b^ | 267.1±32.7^b^ |
| CCL4/MIP-1β | 53.85±7.99^A^ | 247.0±44.5^B^ | 271.1±39.6^B^ | 19.85±0.00^*a^ | 295.3±47.0^b^ | 192.3±18.3^ab^ |
| CCL5/RANTES | 11.93±0.68^A^ | 12.18±0.92^A^ | 12.93±0.65^A^ | 10.17±0.81^a^ | 9.76±0.60^*a^ | 11.09±0.85^a^ |
| CCL11/Eotaxin | 866.9±81.5^A^ | 749.0±43.8^A^ | 774.2±27.6^A^ | 910.6±100.8^*a^ | 885.0±49.0^*a^ | 965.9±62.9^a^ |
| CCL12/MCP-5 | 991.5±77.6^A^ | 3415±442.8^B^ | 3083±427.3^B^ | 778.5±61.2^*a^ | 3122±327.2^b^ | 3086±365.9^b^ |
| CCL17/TARC | 168.9±11.7^A^ | 340.5±13.5^B^ | 285.2±26.1^C^ | 123.7±6.62^*a^ | 395.5±34.1^b^ | 351.5±49.5^b^ |
| CCL19/MIP-3β | 53.23±2.14^A^ | 75.18±3.68^B^ | 71.67±5.66^B^ | 40.02±3.52^*a^ | 73.21±2.86^b^ | 76.74±3.48^b^ |
| CCL20/MIP-3α | 13.60±0.71^A^ | 12.63±0.42^A^ | 13.31±0.67^A^ | 10.47±0.78^a^ | 12.49±1.25^b^ | 14.59±0.67^b^ |
| CCL21/6Ckine | 8212±7850 | 22399±0 | 106.3±0 | 11937±5508 | N.D. | 15019±0 |
| CCL22/MDC | 14.51±0.83^A^ | 35.45±2.44^B^ | 31.23±3.71^B^ | 11.63±0.69^*a^ | 48.83±4.88^*b^ | 42.78±5.84^b^ |
| CXCL1/KC | 82.23±7.40^A^ | 189.7±19.9^B^ | 205.5±22.4^B^ | 76.90±3.25^a^ | 218.0±20.3^b^ | 200.5±22.5^b^ |
| CXCL2/MIP-2 | 122.3±6.7^A^ | 128.2±8.3^A^ | 123.8±11.4^A^ | 145.2±29.0^a^ | 130.1±10.0^a^ | 127.0±6.5^a^ |
| CXCL5/LIX | 502.4±64.3^A^ | 807.1±111.6^A^ | 862.6±162.7^A^ | 1642±120.5^*a^ | 757.7±101.0^b^ | 1773±584.7^ab^ |
| CXCL9/MIG | 391.3±35.8^A^ | 1056±182.3^B^ | 1167±133.8^B^ | 442.5±47.65^a^ | 1672±380.6^b^ | 1983±532.2^b^ |
| CXCL10/IP-10 | 41.20±2.52^A^ | 249.1±39.0^B^ | 285.7±42.4^B^ | 47.37±2.78^*a^ | 239.3±42.9^b^ | 259.3±50.2^b^ |
| CX3CL1/Fractalkine | 1810±162.6^A^ | 1226±67.9^B^ | 1132±107.5^B^ | 1532±120.1^a^ | 1757±159.4^*a^ | 1663±128.7^*a^ |
| EPO | N.D. | N.D. | N.D. | N.D. | N.D. | N.D. |
| G-CSF | 1.82±0.53^A^ | 3.96±1.02^A^ | 4.98±1.14^A^ | 2.24±0.84^a^ | 1.02±0.38^*a^ | 1.83±0.71^a^ |
| GM-CSF | 14.35±0.00^A^ | 52.33±7.09^B^ | 56.61±6.48^B^ | N.D.^*a^ | 40.08±6.66^b^ | 37.71±6.86^b^ |
| IFNβ-1 | 173.8±4.6^A^ | 158.2±4.9^A^ | 147.5±15.4^A^ | 118.6±9.3^*a^ | 159.6±11.9^b^ | 156.7±13.1^ab^ |
| IFNγ | 27.27±1.83^A^ | 22.76±2.78^A^ | 27.01±2.47^A^ | 24.79±2.35^a^ | 26.16±3.15^a^ | 36.46±11.85^a^ |
| IL-1α | 532.7±84.7^A^ | 325.2±23.8^B^ | 348.9±20.5^B^ | 324.4±41.2^a^ | 359.7±16.9^a^ | 398.0±19.1^a^ |
| IL-1β | 14.19±1.33^A^ | 15.70±0.80^A^ | 15.41±1.92^A^ | 12.29±1.01^a^ | 11.86±1.46^*a^ | 15.46±0.55^a^ |
| IL-2 | 33.47±1.86^A^ | 32.66±2.07^A^ | 36.21±1.29^A^ | 35.44±2.20^a^ | 34.03±2.01^a^ | 38.52±1.75^a^ |
| IL-3 | 0.91±0.26^A^ | 0.96±0.25^AB^ | 0.40±0.00^B^ | 142.0±37.9^*a^ | 130.0±28.8^*a^ | 95.0±23.9^*a^ |
| IL-4 | 1.57±0.07^A^ | 1.86±0.11^A^ | 2.23±0.19^B^ | 1.59±0.06^a^ | 8.76±3.94^b^ | 2.36±0.46^ab^ |
| IL-5 | 2.20±0.81^A^ | 1.92±0.95^A^ | 2.67±0.74^A^ | 1.77±0.38^a^ | 2.20±0.72^a^ | 2.27±0.63^a^ |
| IL-6 | 10.34±3.12^A^ | 15.27±1.63^B^ | 16.03±2.21^B^ | 8.21±0.74^a^ | 12.95±1.49^b^ | 10.26±0.92^*ab^ |
| IL-7 | 13.48±1.48^A^ | 12.38±1.16^A^ | 13.91±2.17^A^ | 10.98±1.37^a^ | 10.28±1.41^a^ | 14.59±1.09^a^ |
| IL-9 | 283.2±10.1^A^ | 281.2±25.4^A^ | 289.9±19.6^A^ | 239.7±22.7^a^ | 311.4±8.25^b^ | 320.3±11.0^b^ |
| IL-10 | 33.49±2.29^A^ | 30.68±2.39^A^ | 27.16±3.22^A^ | 31.80±2.74^a^ | 25.99±1.72^a^ | 30.37±3.86^a^ |
| IL-11 | 16.99±1.07^A^ | 23.97±2.44^A^ | 18.91±2.25^A^ | 11.28±0.57^*a^ | 15.53±0.97^*b^ | 16.98±0.97^b^ |
| IL-12p40 | 55.26±6.85^A^ | 46.92±3.82^A^ | 49.83±6.36^A^ | 55.75±3.54^a^ | 37.93±4.08^b^ | 50.16±3.43^ab^ |
| IL-12p70 | 9.95±2.83^A^ | 12.21±3.37^A^ | 4.17±1.96^A^ | 11.28±10.43^*a^ | 6.53±4.76^a^ | 8.42±3.77^a^ |
| IL-13 | 28.97±2.81^A^ | 26.14±2.55^A^ | 34.26±3.55^A^ | 26.08±0.98^a^ | 29.66±1.49^ab^ | 35.89±2.66^b^ |
| IL-15 | 86.33±7.13^A^ | 80.44±8.44^A^ | 74.50±5.66^A^ | 79.20±9.38^a^ | 67.25±4.50^a^ | 70.23±6.77^a^ |
| IL-16 | 1746±263.3^A^ | 1922±351.7^A^ | 2033±55.2^A^ | 1256±376.6^a^ | 2514±222.0^b^ | 2259±247.8^ab^ |
| IL-17 | 0.91±0.10^A^ | 3.50±0.63^B^ | 2.91±0.63^B^ | 0.59±0.11^*a^ | 1.72±0.27^*b^ | 1.64±0.34^b^ |
| IL-20 | 34.75±3.74^A^ | 31.65±3.44^A^ | 45.84±5.58^A^ | 24.83±2.43^*a^ | 34.37±2.20^a^ | 24.87±5.47^*a^ |
| LIF | 5.97±0.29^A^ | 14.19±1.55^B^ | 11.14±1.08^B^ | 4.27±0.49^*a^ | 8.81±0.42^*b^ | 8.13±0.86^*b^ |
| M-CSF | 15.15±1.00^A^ | 24.09±1.51^B^ | 22.94±2.45^B^ | 12.56±1.06^a^ | 27.73±3.39^b^ | 20.83±1.65^b^ |
| TIMP-1 | 443.1±34.6^A^ | 2427±164.8^B^ | 1868±263.2^B^ | 243.6±38.5^*a^ | 1518±167.4^*b^ | 1281±170.1^b^ |
| TNF-α | 7.62±0.43^A^ | 21.24±3.44^B^ | 21.08±3.41^B^ | 7.46±1.00^a^ | 33.26±6.06^b^ | 24.78±3.85^b^ |
| VEGF | 297.6±26.3^A^ | 198.5±31.4^B^ | 238.3±10.7^AB^ | 267.5±32.0^a^ | 216.9±15.6^b^ | 223.3±16.5^b^ |

Data are presented as pg cytokine per g of lung tissue (mean ± SEM, n = 8/gp). Differences between VEH/CON, cSiO_2_/CON, and cSiO_2_/TPPU groups within the 7d PI and 28d PI cohorts were compared by Student’s t test. Differences between the 7d PI and 28d PI VEH/CON, cSiO_2_/CON, and cSiO_2_/TPPU groups were also compared by Student’s t test. Alternative versions of this test was used when data did not meet the assumption of normality and/or equal variances. Asterisks indicate significant differences between the VEH/CON 7d PI and VEH/CON 28d PI groups, cSiO_2_/CON 7d PI and cSiO_2_/CON 28d PI groups, or cSiO_2_/TPPU 7d PI and cSiO_2_/TPPU 28d PI groups (p<0.05). Unique uppercase letters indicate significant differences between the VEH/CON 7d PI, cSiO_2_/CON 7d PI, and cSiO_2_/TPPU 7d PI groups (p<0.05). Unique lowercase letters indicate significant differences between the VEH/CON 28d PI, cSiO_2_/CON 28d PI, and cSiO_2_/TPPU 28d PI groups (p<0.05).


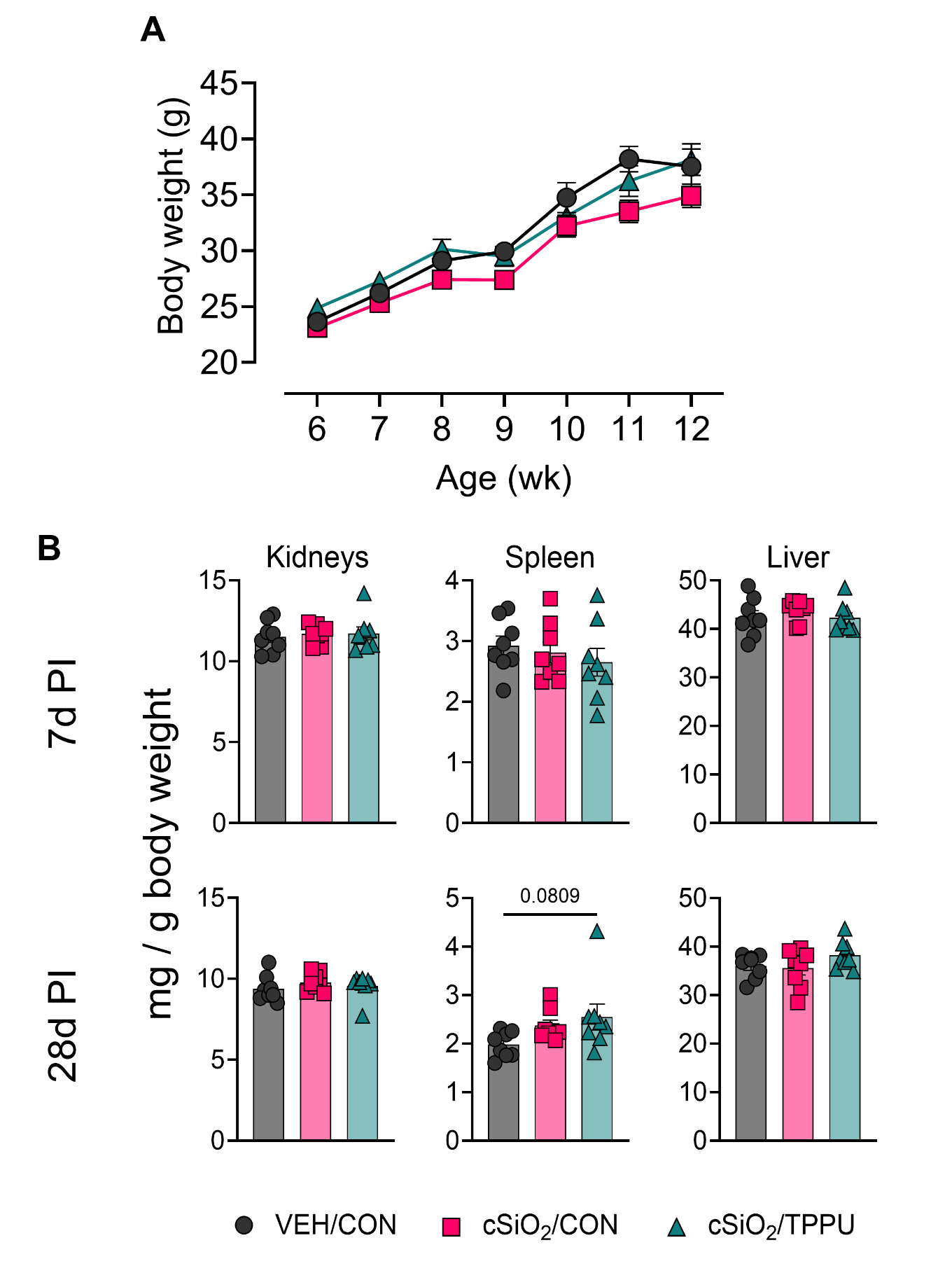


**Supplementary Figure 1. Acute cSiO_2_ exposure and dietary TPPU supplementation do not significantly impact growth rate and post necropsy kidney, spleen, and liver weights in female autoimmune-prone NZBWF1 mice. (A)** In both cohorts, body weights were monitored weekly. Data from the 7d PI and 28d PI cohorts were pooled from 6-9 wk of age. cSiO_2_ and TPPU did not significantly influence total body weight during the entire study. **(B)** At 7d PI and 28d PI, cohorts of NZBWF1 mice were sacrificed, and wet organ weights for both kidneys, spleen, and liver were measured prior to downstream tissue processing. Data are presented as mean ± SEM. Values of p<0.1 are shown, with p<0.05 considered statistically significant.
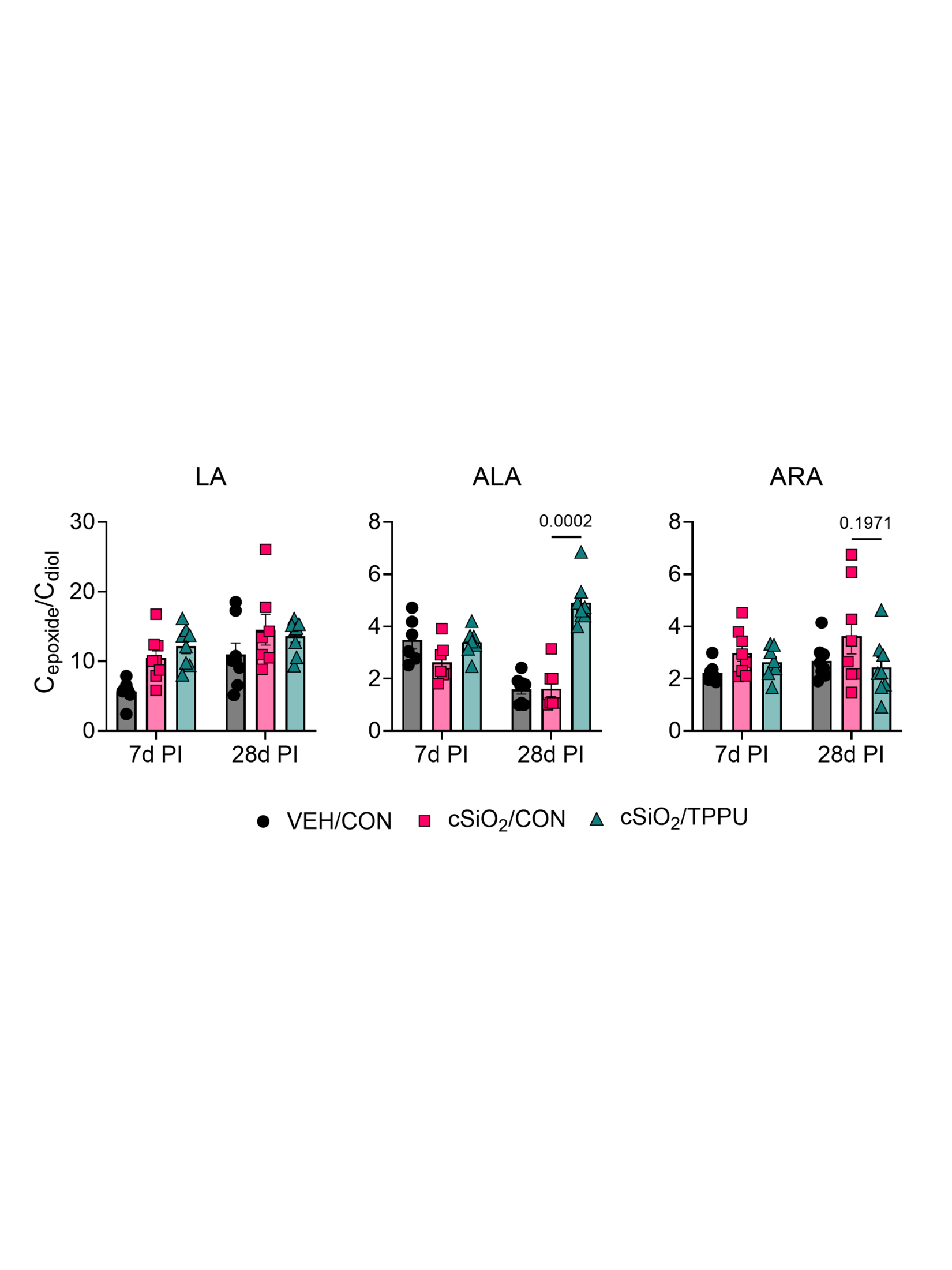


**Supplementary Figure 2. Dietary TPPU supplementation does not significantly increase sEH metabolites in the lung.** Except for ALA-derived metabolites at 28d PI, TPPU consumption did not significantly alters epoxide/diol ratios for metabolites derived from LA, ALA, and ARA in lung. Ratios between sums of all EpFAs and sums of all DiHFAs from each PUFA precursor are shown. Data are presented as mean ± SEM (n = 8). <LOD; below limit of detection. Values of p<0.2 are shown, with p<0.05 considered statistically significant.


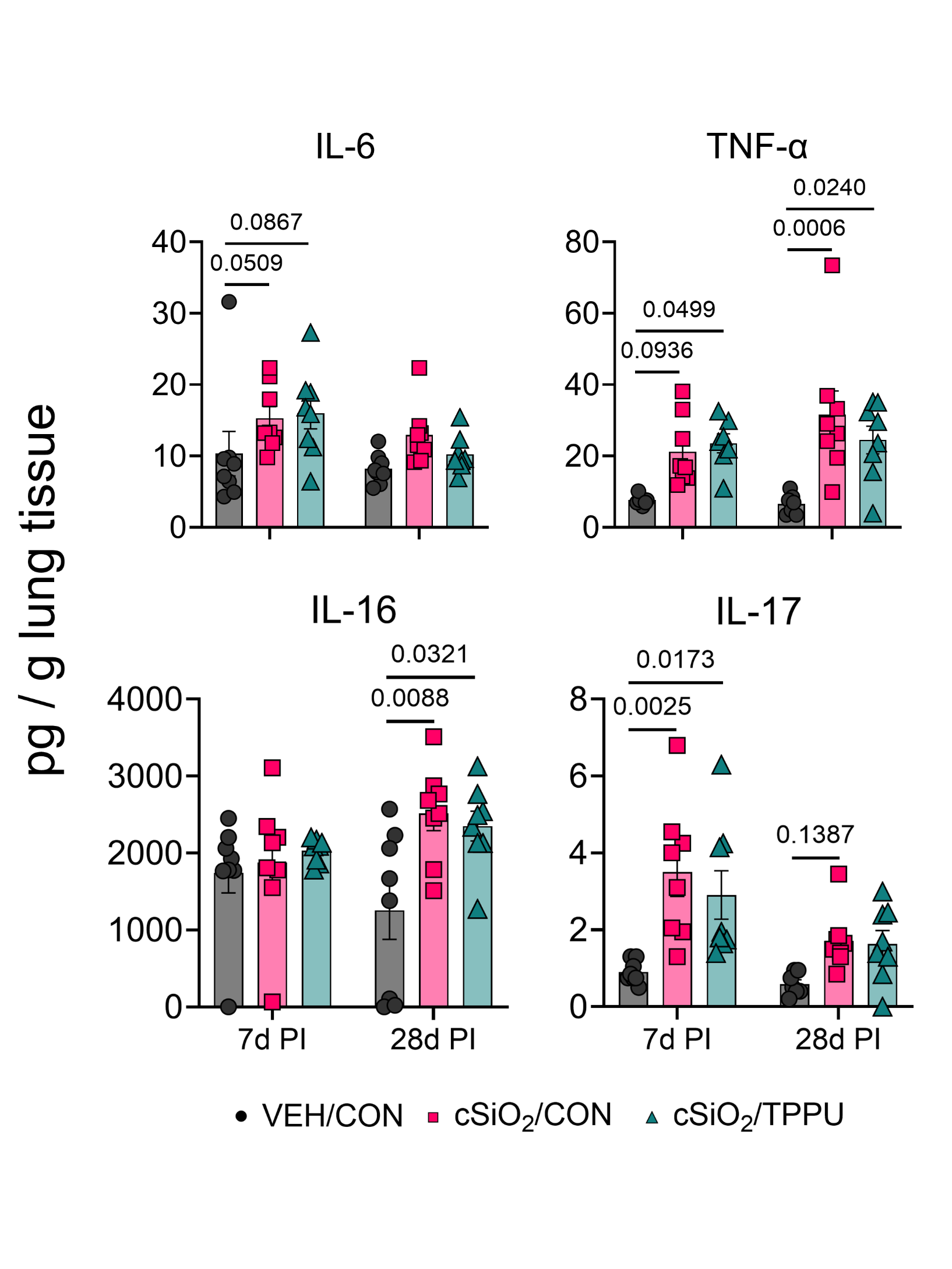


**Supplementary Figure 3. cSiO_2_ induces production of inflammatory and adaptive cytokines in the lung.** Following sacrifice, middle lung lobes were isolated and homogenates analyzed for production of selected cytokines (i.e., IL-6, TNF-α, IL-16, IL-17) using Mouse Cytokine/Chemokine 44-Plex Discovery Assay® Array from Eve Technologies. Cytokine quantities were normalized to the original weight of lung tissue homogenized for the analysis. For individual data points that fell below the limit of detection, LOD/2 was substituted for statistical analysis. Data are presented as mean ± SEM (n = 8). Values of p<0.2 are shown, with p<0.05 considered statistically significant.
